# The Mighty Mitochondria Are Unifying Organelles and Metabolic Hubs in Multiple Organs of Obesity, Insulin Resistance, Metabolic Syndrome, and Type 2 Diabetes: An Observational Ultrastructure Study

**DOI:** 10.3390/ijms23094820

**Published:** 2022-04-27

**Authors:** Melvin R. Hayden

**Affiliations:** Endocrinology Diabetes and Metabolism, Diabetes and Cardiovascular Disease Center, Department of Internal Medicine, University of Missouri School of Medicine, One Hospital Drive, Columbia, MO 65211, USA; mrh29pete@gmail.com; Tel.: +57-33-463-019

**Keywords:** aberrant mitochondria, mitochondria, insulin resistance, type 2 diabetes mellitus, mtDNA, mtROS, fusion, fission, biogenesis, mitophagy

## Abstract

Mitochondria (Mt) are essential cellular organelles for the production of energy and thermogenesis. Mt also serve a host of functions in addition to energy production, which include cell signaling, metabolism, cell death, and aging. Due to the central role of Mt in metabolism as metabolic hubs, there has been renewed interest in how Mt impact metabolic pathways and multiple pathologies. This review shares multiple observational ultrastructural findings in multiple cells and organs to depict aberrant mitochondrial (aMt) remodeling in pre-clinical rodent models. Further, it is intended to show how remodeling of Mt are associated with obesity, insulin resistance, metabolic syndrome (MetS), and type 2 diabetes mellitus (T2DM). Specifically, Mt remodeling in hypertensive and insulin-resistant lean models (Ren2 rat models), lean mice with streptozotocin-induced diabetes, obesity models including diet-induced obesity, genetic leptin-deficient *ob/ob*, and leptin receptor-deficient *db/db* diabetic mice are examined. Indeed, aMt dysfunction and damage have been implicated in multiple pathogenic diseases. Manipulation of Mt such as the induction of Mt biogenesis coupled with improvement of mitophagy machinery may be helpful to remove leaky damaged aMt in order to prevent the complications associated with the generation of superoxide-derived reactive oxygen species and the subsequent reactive species interactome. A better understanding of Mt remodeling may help to unlock many of the mysteries in obesity, insulin resistance, MetS, T2DM, and the associated complications of diabetic end-organ disease.

## 1. Introduction

**Figure 1:**
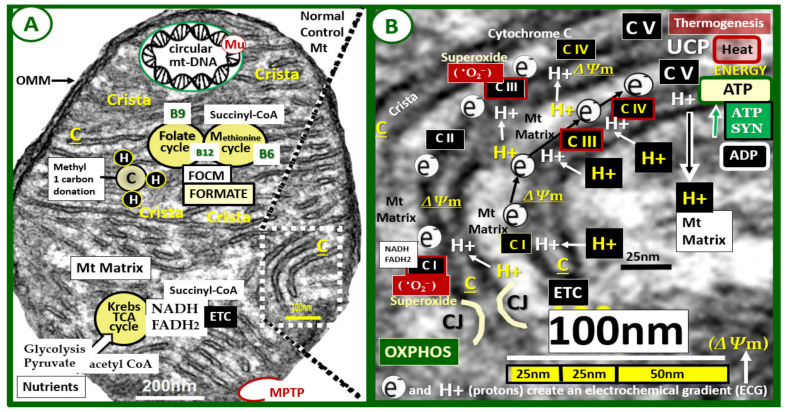
Normal mighty mitochondria (Mt). These images demonstrate the normal electron transport chain (ETC) and adenosine triphosphate (ATP) production in a 20-week-old healthy, control C57BL/6J female mouse model. **Panel A** demonstrates an exploded image of a normal mitochondrion with its numerous cristae (C) (invaginations of the inner membrane to increase its surface area). Note the Krebs—TCA cycle lower-left that utilizes acetyl CoA from either glycolysis or fatty acid metabolism and importantly donates NADH and FADH2 to enter the electron transport chain (ETC) at complex I (CI) in **panel B**. Additionally, note the folate and methionine cycles representing the Mt folate-mediated one-carbon metabolism (FOCM) with their essential vitamin cofactors B12, B9, and B6. Importantly, formate is supplied to the nucleus via FOCM as well as succinyl-CoA from the methionine cycle (M) and also donates methyl groups to mtDNA, and nuclear DNA for maintenance and repair. Importantly, note that mitochondria have their own circular DNA that is capable of undergoing mutation(s) (Mu). Additionally, note the presence of a mitochondria permeability transition pore (MPTP) lower-right in red coloring. **Panel B** is an exploded image of the boxed-in (white dashed lines) in **panel A**, which depicts an even higher exploded magnification (scale bar 100 nm) to improve the clarity of the Mt inner membrane cristae structures and the ETC. Note the movement or flow of electrons (e^−^) from complex I to complex IV and hydrogen protons (H^+^), which create the mitochondrial membrane potential (ΔΨm) generated by the proton-pumping action from the matrix to the inner membrane space at complexes I, III, and IV. This results in the formation of ATP (energy) at complex V as protons (H^+^) flow back into the Mt matrix through complex V resulting in the subsequent formation of ATP (energy) at complex V via the ADP substrate and ATP SYN [8]. The ETC may become overwhelmed when there is nutrient excess, such as occurs in hyperglycemia and hyperlipidemia associated with obesity and T2DM. Additionally, uncoupling protein(s) (UCPs), located on the inner Mt membrane are known to uncouple oxygen consumption by the respiratory chain from ATP synthesis, and it should be noted that thermogenesis (non-shivering) upper-right occurs in brown adipose tissue mitochondria that stain positive for uncoupling protein1 (UCP1) [8]. Original magnification ×12,000 and scale bars = 200 nm (**panel A**) and 100 nm (**panel B**). ADP = adenosine diphosphate; ATP SYN = adenosine triphosphate synthase; C = complex; CJ = crista junction; CoA = coenzyme A; FADH2 = reduced flavin adenine dinucleotide; NADH = reduced nicotinamide adenine dinucleotide; OMM = outer mitochondrial membrane; ^•^O_2_^−^ = superoxide; OXPHOS = oxidative phosphorylation; TCA = tricarboxylic cycle.

Mitochondria (Mt) are highly dynamic and mobile cellular organelles that contribute to homeostasis by maintaining energy for cells to function in the form of adenosine-5′-triphosphate (ATP) [1,2,3,4,5,6,7,8]. They are unifying organelles and metabolic hubs, which ultimately determine cellular homeostasis for survival, and cellular death when they are remodeled and damaged to an aberrant phenotype. Further, a decrease or loss of ATP from Mt places cells in great danger of survival. Specifically, Mt serve to produce energy from the tricarboxylic acid (TCA) or Krebs cycle and fatty acid beta-oxidation in the electron transport chain (ETC) in the form of ATP, which are essential for proper cellular functioning and life. Additionally, Mt generate low levels of reactive oxygen species (ROS) in the form of superoxide via the electron transport chain (ETC) that are important for physiologic cell signaling and maintaining cellular homeostasis (Figure 1) [1,2,3,4,5,6,7,8].

**Figure 2:**
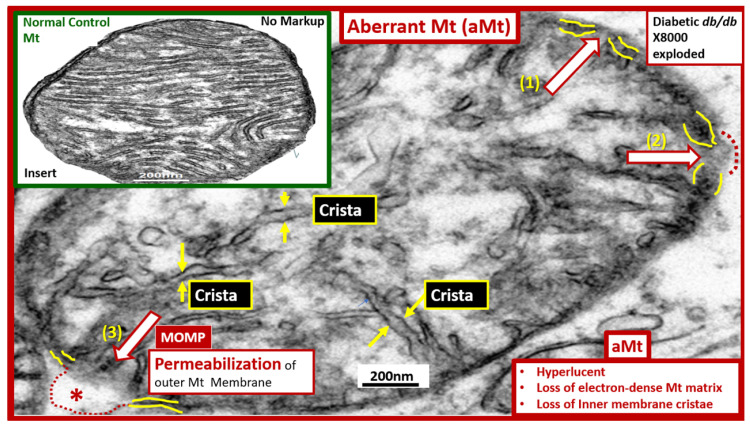
Aberrant mitochondria (aMt). This image depicts an exploded aberrant mitochondrion with an intact scale bar from a 20-week-old obese, insulin-resistant, diabetic *db/db* female mouse model. aMt are a common phenotype found in multiple different cells and organs in models of obesity, insulin resistance, metabolic syndrome, and type 2 diabetes mellitus as compared to control healthy C57BL/6J models (insert upper left from Figure 1A). The mitochondria organelles, along with the nucleus are known to be contained by outer and inner membrane structures similar to the cellular plasma membrane (fine yellow lines of the mitochondrion outer and inner membranes). This aberrant mitochondrion depicts the ultrastructural remodeling that is found to be present in almost all of the subsequent TEM images. aMt may be characterized by their hyperlucency, loss of cristae (yellow arrows), loss of electron-dense mitochondrial matrix, and loss or disruption of the outer membranes (red open arrows). aMt are known to be leaky with disruption or loss of the Mt outer membranes as depicted in (1) thinning of the inner and outer membranes (open red arrow); (2) interruption of the inner and outer membranes with partial permeabilization (open red arrow and red dashed line); (3) complete loss of the inner and outer membranes with permeabilization of the outer membrane (open red arrow, dashed red lines, and asterisk). Importantly, the mitochondrial outer membrane permeabilization (MOMP) allows the contents of the Mt to leak damaging superoxide—mitochondria reactive oxygen species (mtROS), which are capable of reacting with nitrogen, and sulfur species from the iron-sulfur complexes known to be present in complex I and the cytosol to form a reactive species interactome (RSI) consisting of reactive oxygen, nitrogen, sulfur species (RONSS), and the apoptotic death-promoting protein cytochrome c. Additionally, MOMP allows the leakage of mtDNA that can activate damage-associated molecular patterns (DAMPs) that are capable of initiating a sterile inflammatory response by binding to pattern-recognition receptors. Importantly, MOMP is considered to be a ‘point of no return’ as this event is responsible for engaging the apoptotic cascade in numerous cell death pathways. The development of MOMP is thought to be associated with the pro-apoptotic BCL-2-associated X protein (BAX) or B-cell lymphoma-2 (BCL-2) antagonist or killer (BAK). However, it is important to also note that an increase in nutrient excess and resultant mtROS may damage the outer membrane lipoproteins via mtROS induced lipid peroxidation, propagation of lipid peroxidation chain reactions, and increase the opportunity for MOMP to occur from the “inside-out” to damage the outer Mt membrane. Additionally, the emerging role of activated mitochondrial matrix metalloproteinases (mtMMPs) may also play a role in outer membrane permeabilization. This aberrant phenotype results in a loss of function of the electron transport chain responsible for the production of cellular energy—adenosine triphosphate (ATP). Importantly, if there is an impairment in mitophagy, leaky aMt via mitochondria permeability transition pores (MPTPs) and MOMPs will accumulate in cells and contribute to ongoing cellular dysfunction and damage due to ATP depletion and eventually cellular apoptosis via cytochrome c. Original magnification ×8000; with intact scale bar = 200 nm. TEM = transmission electron microscopy.

These dynamic organelles also have a highly adaptive capacity to respond to physiologic stressors, especially metabolic stressors, in the form of Mt fusion, fission, biogenesis, and mitophagy. However, these adaptive capacities of the Mt can be exceeded due to chronic stressors such as nutrient excess in obesity and T2DM and develop aberrant Mt (aMt) with defective changes in morphology and phenotypes that are associated with multiple disease states and especially in obesity, IR, MetS, and T2DM (Figure 2).

**Figure 3:**
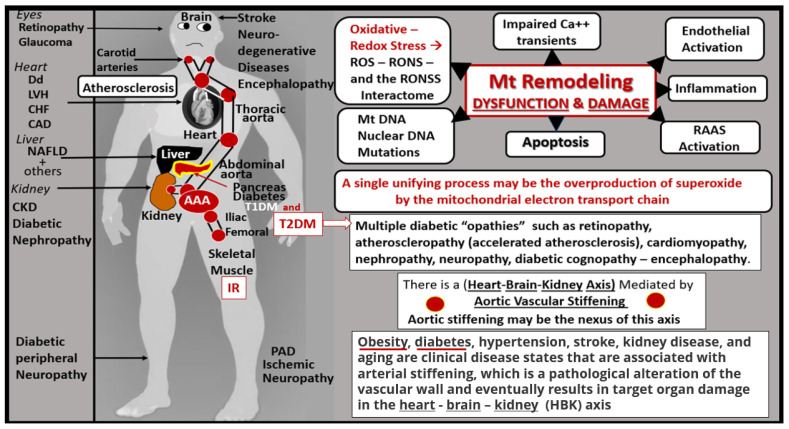
Mitochondria (Mt) remodeling to an aberrant Mt (aMt) phenotype with dysfunction and damage to organ systems. This figure is an illustration of proposed mitochondrial-mediated mechanisms of disease development and progression associated with aMt dysfunction and damage that have been implicated in numerous organ systems and clinical diseases (left-hand standing man image). Oxidative—redox stress includes the sum of reactive oxygen species (ROS), reactive nitrogen species (RNS), and reactive sulfur species (RSS) to create the reactive oxygen, nitrogen, sulfur species (RONSS) interactome. Importantly, aortic stiffening may be the nexus for a heart-brain-kidney axis (red circles) illustrating the aortic vascular pathway of vascular stiffening to affect the carotid and renal arteries and target organs of the heart-brain-kidney axis. AAA = abdominal aortic aneurysm; Ca++ = calcium transients; CAD = coronary artery disease; CHF = congestive heart failure; CKD = chronic kidney disease; Dd = diastolic dysfunction; DNA = deoxyribonucleic acid; IR = insulin resistance; LVH = left ventricular hypertrophy; NAFLD = non-alcoholic fatty liver disease; PAD = peripheral arterial disease; RAAS = renin-angiotensin-aldosterone system; T1DM = type 1 diabetes mellitus; T2DM = type 2 diabetes mellitus.

Importantly, dysfunction and damage to Mt result in decreased ATP generation and the excessive generation of Mt superoxide—Mt ROS (mtROS), which are capable of propagating cellular dysfunction or loss via apoptosis that can result in multiple organs, which can result in multiple human pathologies (Figure 3).

Mt have a variety of sizes, shapes, and structures, and they are functionally complex and intimately integrated with many aspects of cellular metabolism [1,2,3,4,5,6,7,8]. Mt are often depicted as discrete organelles; however, they form a reticular-tubular type of network within cells depending on cell type and environment and interact intimately with the endoplasmic reticulum and nuclear membranes. The interconnected reticular network or threaded granules of Mt are not stationary but capable of moving about the cell to where energy needs may be the greatest and where cellular signaling by Mt are necessary [9,10,11]. This movement of Mt is supported by the complex Mt interaction with molecular motors along the actin cytoskeleton and microtubule networks that are also dependent on the energy production by the Mt [9,10,11]. Mt contain two ribosomal and 22 transfer RNA genes that are required for mitochondrial protein synthesis and 13 essential protein-encoding genes from the circular mitochondria deoxyribonucleic acid (mtDNA) that are necessary for the formation of ETC complexes (I, III, IV, V) [7,8,9]. It is important to note here that complex II proteins are encoded by nuclear DNA [12].

The ETC within the Mt is a complex interaction and may be described as follows: Mitochondria generate energy from electrons that are passed from their donors at lower redox potential to acceptors at higher redox potential through the ETC of its complexes (I–IV). Along with this electron movement, H+ protons are pumped from the matrix to the inner membraneous space that generates an energy potential difference across the inner membrane. This energy potential (preserved mitochondrial membrane potential (Δ*Ψ*m)) is then transferred to ATP from adenosine diphosphate (ADP) or dissipated as heat (non-shivering thermogenesis via UCP) as H+ protons leak back toward the matrix of the ETC complex (V) [8]. In this process, most of the electrons are eventually passed to molecular oxygen and a small portion is leaked during this transport. This mechanism results in the one-electron reduction of oxygen to superoxide. The superoxide (^•^O_2_^−^) generating ROS (mt-ROS) may become destructive to the cell due to free radical damage in response to nutrient excess; however, at low levels of production, superoxide serves as a metabolic cell signaling mechanism. The ETC becomes overwhelmed when there is nutrient excess such as hyperglycemia and hyperlipidemia associated with T2DM. Mt-derived superoxide is capable of interacting with the reactive species interactome [13,14], which includes not only mt-ROS but also reactive nitrogen species and reactive sulfur species such as occurs in the presence of iron-sulfur clusters in complex I or the reactions when there is impaired folate one-carbon metabolism and hyperhomocysteinemia (HHcy) that form reactive species due to homocysteine (Hcy) autoxidation, mixed disulfide formation, the interaction of reactive Hcy thiolactones, and homocysteinylation [15]. Thus, these affected cells will experience damage associated with increased oxidative-redox stress of the reactive species interactome (RSI) [13]. This RSI is an interactome consisting of functional interactions between molecules within a cell due to a combination or sum of reactive oxygen, nitrogen, and sulfur species (RONSS) [11,12,13].

**Figure 4:**
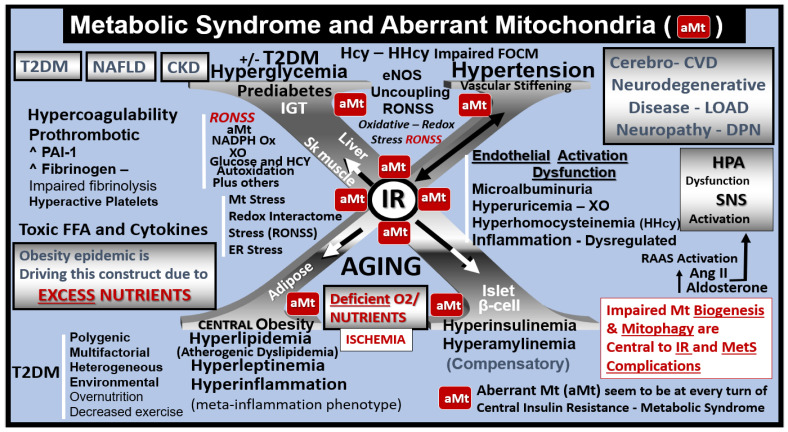
The metabolic syndrome (MetS) and aberrant mitochondria (aMt). This image illustrates how the complex MetS and aMt can have a tremendous interaction and impact with multiple crosstalk as they intersect. In its simplest form, the MetS may be characterized by the clustering of multiple metabolic abnormalities and clinical disease states associated with multiple vulnerabilities. The central “X” in this figure honors Jerry Reaven [18] who initially coined the term Syndrome X and championed the concept that the resistance to insulin-mediated glucose disposal—insulin resistance (IR) was a characteristic of patients with type 2 diabetes mellitus (T2DM) and cerebrocardiovascular disease (CVD), which was later termed MetS by Scott Grundy [19]. There are four arms to this letter X and each arm has a designated condition to further illustrate the “H” phenomenon, representing a “hyper” state, e.g., hyperlipidemia, lower left; pancreatic islet β-cell hyperinsulinemia/hyperamylinemia, lower right; hypertension and vascular stiffening, upper right; hyperglycemia, upper left. Note how IR is placed central to each of the four arms and how it is surrounded by aMt, which represents Mt dysfunction and damage [17]. Each of the four arms is important and note the arrows depicting that IR can impact each of the tissues and the clinical disease states. Further, note that the central IR is surrounded by the red coloring of the aMt that are so important to this review. Additionally, the MetS constitutes a milieu conducive to tissue oxidative-redox stress. Oxidative-redox stress incorporates the sum of reactive oxygen, nitrogen, and sulfur species stress (RONSS) and its cellular RONSS interactome and inflammation that create a vicious cycle. The multiple metabolic toxicities associated with the MetS including the RONSS interactome and endothelial dysfunction combine to weave a complicated mosaic fabric, which is also known to be associated with the activation of the renin-angiotensin-aldosterone-system (RAAS). Additionally, cerebrocardiovascular disease (CVD), which includes neurodegenerative disease such as late-onset Alzheimer’s disease (LOAD) and/or a diabetic encephalopathy and chronic kidney disease (CKD) together comprising the heart–brain-kidney axis that is involved when there is aortic vascular stiffening associated with MetS and T2DM. It is important to note that hyperinsulinemia is initially protective and a compensatory response to nutrient overload; however, if hyperinsulinemia due to IR remains chronic there is a price to pay by becoming a central commonality along with the development of aMt and the development of RONSS interactome and its detrimental role in each of the four arms of the syndrome X and its clinical disease states and syndromes. The addition of aMt adds even further strength to understanding the important role of the MetS since Mt function plays a key role in IR in general and even more so in T2DM. Importantly, homocysteine (Hcy) and hyperhomocysteinemia (HHcy) could be added to the “H” phenomenon, since HHcy is an independent risk factor for cerebrocardiovascular disease (CVD) and hypertension and vascular stiffening in addition to being a biomarker for impaired folate one-carbon metabolism. AGE = advanced glycation end products; Ang II = angiotensin II; CKD = chronic kidney disease; CVD = cerebrocardiovascular disease; eNOS = endothelial nitric oxide synthase; ER = endoplasmic reticulum; ESRD = end-stage renal disease; FFA = free fatty acids; FOCM = folate-mediated one-carbon metabolism; HPA = hypothalamic–pituitary–adrenal axis; LOAD = late-onset Alzheimer’s disease; Mt = mitochondria; NADPH Ox = nicotinamide adenine dinucleotide phosphate oxidase; NAFLD = non-alcoholic fatty liver disease; NASH = non-alcoholic steatohepatitis; O_2_ = oxygen; PAI-1 = plasminogen activator-1; RAGE = receptor for AGE; SNS = sympathetic nervous system; XO = xanthine oxidase.

The intersection of the MetS and aberrant mitochondria (aMt) may not only have immediate but also long-term consequences in regard to the constellation of metabolic abnormalities and clinical disease states with multiple organ vulnerabilities associated with age-related diseases (Figure 2, Figure 3 and Figure 4) [14,15,16,17,18,19].

The dynamic, shape-shifting morphology and functional complexity of Mt are not to be underestimated. This review demonstrates that multiple different organs and cells have the capability for the Mt to undergo a rather remarkable remodeling of their structure not only in their phenotypic morphology but also in their function. The primary functional mechanistic studies of Mt are extremely necessary and vitally important to the contribution of the abnormally remodeled aMt detected by transmission electron microscopy (TEM). However, TEM still remains the ultimate gold standard for the study of Mt and their precise morphology and their varying ultrastructural phenotypes in different disease states in multiple cells and organs. Once the Mt become aberrantly remodeled to an aMt phenotype and impaired mitophagy is present, they contribute mechanistically via mtROS and the reactive species interactome to the ongoing cellular dysfunction and damage.

Importantly, Mt are known to be susceptible to different genetic and environmental injuries, the accumulation of mtDNA mutations, and mtDNA copy number depletion, and the epigenetic modification of the Mt genome may help to explain the prevalence of Mt-related diseases including (T2DM) [7,9,20].

## 2. Brief Overview of Mitochondrial Dynamics (Fusion and Fission), Biogenesis, and Mitophagy

**Figure 5:**
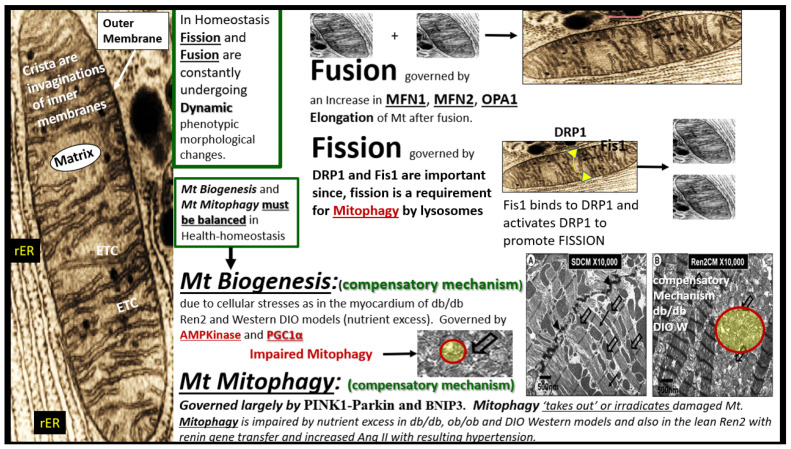
Possible morphological remodeling mechanisms as described by a brief overview of mitochondrial (Mt) fusion, fission, biogenesis, and mitophagy. Mt are known to consist of four distinct compartments: the outer membrane (arrow), the intermembrane space (not shown), the inner membrane of the crista, and the matrix (far left-hand side of this image). In general Mt consist of various shapes and sizes ranging from 0.5–3 μm with an outer membrane of ~7 nm and an inner membrane of ~5 nm. In living cells mitochondria are constantly in motion and moving, changing their shapes and sizes, and are observed to fuse (fusion) or split (fission). The adaptive processes of Mt fusion, fission, biogenesis, and mitophagy are illustrated in this figure with a brief description of the governing mechanisms and images. As one begins to better understand these complex mechanisms that are in play in various diseases the better, we become at understanding Mt remodeling in their response to injury to various metabolic stressors. AMP Kinase = adenosine monophosphate-activated protein kinase; Ang II = angiotensin II; BNIP3 = Bcl-2 (B-cell lymphoma 2)/adenovirus E1B 19-kDa-interacting protein 3; DIO = diet-induced obesity; DRP1 = Dynamin-related protein 1; ETC = electron transport chain; Mt = mitochondria; MFN1 and 2 = mitofusin 1 and 2; OPA1 = optic atrophy gene 1; Parkin = PTEN (Phosphatase and tensin homolog)—induced kinase 1; PINK = Parkinson’s disease-associated gene; PTC = proximal tubule cell; PGC1α = peroxisome proliferator-activated receptor-gamma coactivator; W = Western diet-induced obesity; rER = rough endoplasmic reticulum.

Before examining the Mt in each of the individual organs, it is necessary to briefly identify each of the Mt remodeling mechanisms as it pertains to fusion, fission, biogenesis, and mitophagy (Figure 5).

The previous figure illustrates the possible various morphological mechanisms and remodeling of Mt in health and disease. The functional mechanisms are extremely complex and much has been learned about fusion, fission, biogenesis, and mitophagy with the use of genetic knockout/knockin models. For a more complete discussion of the complicated interactions regarding the functional molecular mechanisms, the following reference is highly recommended [20].

It is also important to note that extramitochondrial oxygen consumption also occurs via nonenzymatic and other enzymatic reactions, which are derived from NADPH oxidase, xanthine oxidase, uncoupled nitric oxide synthase, D-amino oxidase, p450 cytochromes, and proline hydroxylases [20]. In the following sections, multiple cells from different organs are examined to demonstrate how normal Mt are remodeled to an aMt phenotype in obesity, insulin resistance, MetS, and T2DM.

## 3. Myocardial Remodeling: Increased Mitochondrial Biogenesis, Impaired Mitophagy, and Accumulation of Aberrant Mt (aMt) in Obesity, IR, MetS, and T2DM

The myocardium is very unique because it requires a constant source of ATP in order to provide adequate continuous myocardial contraction and this specialized muscle must be able to increase its ATP production for any increase in heart rate or respond to an increased end-diastolic pressure to promote homeostasis [16,21,22,23]. Importantly, diabetic cardiomyopathy (DC) that occurs in T2DM is associated with dysfunctional Mt (aMt). DC may be considered a condition of myocardial dysfunction in the absence of overt clinical coronary artery disease, valvular disease, and other conventional cardiovascular risk factors, such as hypertension and dyslipidemia that occur with diabetes. DC may be characterized by multiple mechanisms, which include: mitochondrial dysfunction (aMt), disruption in cardiac insulin signaling, increased oxidative stress, inflammation, decreased nitric oxide bioavailability, elevated advanced glycation end products, accumulation of extracellular matrix (ECM) (fibrosis), ECM and cardiomyocyte stiffening, impaired calcium handling of Mt and cardiomyocytes, endoplasmic reticulum stress, cardiac autonomic neuropathy, microvascular dysfunction, renin–angiotensin–aldosterone system (RAAS) activation, and importantly, the emerging and novel exosome pathways that are now being considered [21,22]. Indeed, healthy Mt are critical for the mechanical function of cardiomyocytes [23].

**Figure 6:**
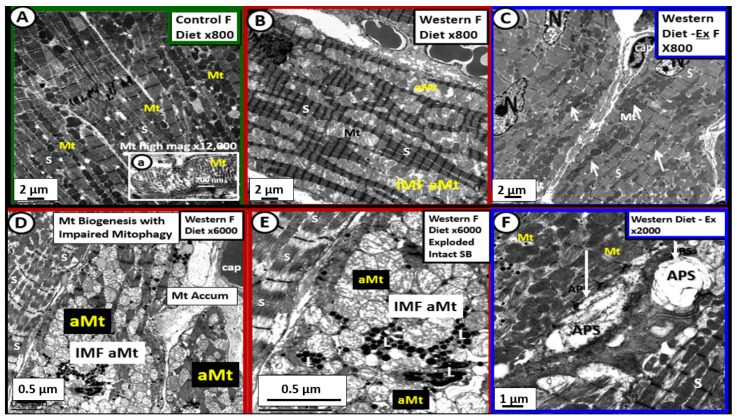
Mitochondrial biogenesis and the accumulation of aberrant mitochondria (aMt) due to impaired mitophagy in the left ventricle of Western diet-induced obesity female mice at 20-weeks of age. **Panel A** illustrates the normal morphology of the alternating row of sarcomeres with usually a single row of electron-dense intermyofibrillar mitochondria (IMF Mt). Note that insert (**a**) depicts normal crista morphology. **Panel B** depicts the moderate expansion of the IMF aberrant Mt (aMt) in Western models with hypertrophic Mt and increased hyperlucency as compared to the electron-dense mitochondria in **panel A**. **Panel C** reveals the protective role of enhanced exercise (Ex) via increased heme oxygenase-1 on Mt remodeling. **Panels D** and **E** depict marked IMF Mt expansion (biogenesis) with an accumulation of aMt that are hyperlucent with attenuation and loss of crista and a loss of the electron-dense Mt matrix in obese Western models. Note that **panel E** is an exploded view of the IMF aMt in **panel D** to better demonstrate the abnormalities of the IMF aMt. **Panel F** is interesting as it depicts areas of mitophagy with 2 different autophagosomes (APS white arrows) amongst areas that appeared more like the control models in **panel A** and in the exercise (Ex) model (**panel C**), which suggests that exercise may improve mitophagy and limit aMt. These modified images were provided with permission by CC 4.0 [25]. Magnifications vary and scale bars are located in the lower left-hand and magnification in the upper right-hand side of each image. Cap = capillary; N = nucleus.

**Figure 7:**
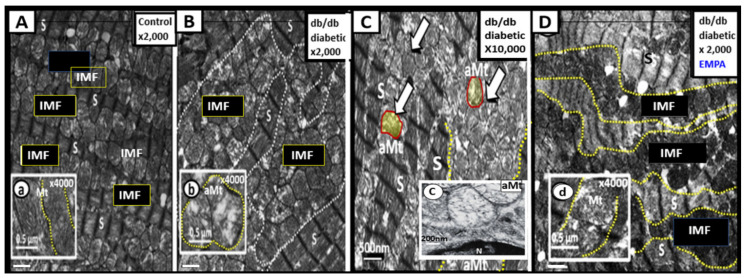
Mitochondria (Mt) biogenesis with impaired mitophagy in the 16-week-old obese, insulin-resistant, female diabetic *db/db* mice. **Panel A** illustrates a row of sarcomeres alternating with a row of intermyofibrillar (IMF) Mt in the lean non-diabetic control model. **Panels B** and **C** depict extensive Mt expansion due to Mt biogenesis with sarcomere disarray and accumulation of aberrant Mt (aMt) (open arrows and encircled yellow with red line). Note the presence of numerous hyperlucent aMt with loss of electron-dense Mt matrix and attenuation of crista as depicted in inserts (**b**) and (**c**) as compared to the control model in insert (**a**). These aMt are representative of impaired mitophagy, which allow aMt to accumulate. Additionally, these aMt are leaky and release increased superoxide–mtROS and cytochrome c, which in turn is associated with cardiomyocyte dysfunction and increased cardiomyocyte apoptosis. **Panel D** demonstrates an improved sarcomere (S) arrangement and note the marked improvement of electron-dense mitochondria in insert (**c**). Additionally, note the improvement of the Mt expansion and note that in insert (**d**) the Mt are more electron-dense and appear similar to control models in insert (**a**) in those models treated with empagliflozin (a sodium glucose transporter-2 inhibitor) for five weeks. These modified images were provided with permission by CC 4.0 [26]. Magnification ×2000 in **panels A**, **B** and **D**; scale bar = 1 μm and Magnification ×10,000; scale bar = 500 nm in **panel C**. Inserts = 0.5 μm in panels (**a**), (**b**), (**d**); scale bars = 200 nm in insert (**c**).

**Figure 8:**
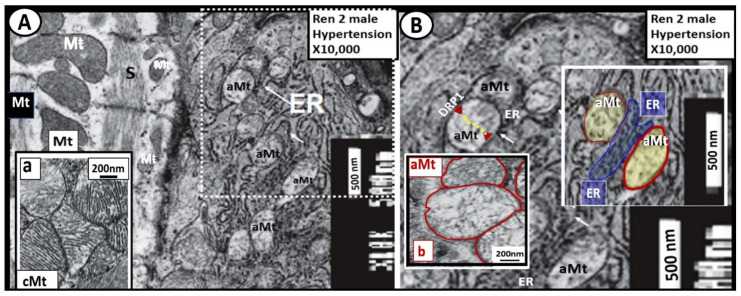
The close ultrastructural relationship between the myocardial mitochondria (Mt) and the endoplasmic reticulum (ER) in the hypertensive 7-week-old male Ren2 model with insulin resistance and impaired glucose tolerance. **Panel A** demonstrates that cardiomyocyte Mt are actually in very close contact with the ER (arrows), which would enable considerable crosstalk in the white dashed boxed-in region. Importantly, note the normal electron-dense Mt in the far left-hand side of this image and insert (**a**) and compare them to the aberrant Mt (aMt) (electron hyperlucent Mt with loss of crista) in **panel B**. **Panel B** depicts a higher magnified exploded view from the dashed boxed-in region of **panel A**. Note the extremely electron-dense membranes of the endoplasmic reticulum (ER) and how the outer membranes of the ER appear to be touching and possibly communicating with the outer membranes of the aberrant Mt (aMt) termed mitochondria-associated membrane(s) or (MAMs). Further, note that the aMt (outlined in red) are in close proximity and apposition with the ER membranes (pseudo-colored blue). This indicates, at the very least, an ultrastructural potential for ER-Mt crosstalk due to MAMs. There is close proximity of less than 100 nm to the actual contact between the outer membranes of the aMt and the ER. This close apposition and touching of the MAMs may allow for considerable crosstalk between the ER and Mt and thus allow for calcium and reactive oxygen species transfer, which may increase ER stress when there is Mt dysfunction due to aMt since the ER utilizes ATP from the Mt. Importantly, impaired Mt fission and mitophagy machinery in concert with ER stress may result in cellular apoptosis. This close apposition/touching allows MAMs to be responsible for (1) calcium buffering; (2) Mt fission; (3) phospholipid exchange. Importantly, the longer these aberrant Mt are not ‘taken-out’ or removed by mitophagy the greater the possibility for reactive oxygen species (mt ROS) and calcium to leak and cause further generation of reactive oxygen, nitrogen, and sulfur species to active the reactive species RONSS interactome and cardiomyocyte dysfunction and Mt leakage of cytochrome c to induce myocardial cellular apoptosis. While this image is from the Ren2 hypertensive myocardium experiment [27], this close association between the ER and aMt is known to be present in other organs in multiple different disease states. Original magnification = ×10,000; scale bars = 500 nm. DRP1 = dynamin-related protein 1 necessary for Mt fission.

The myocardium must also be prepared to rapidly adapt to stressors such as hypertension and increased demands to work against an increased stiffened vascular system that occurs when there is systemic vascular stiffening and increased myocardial end-diastolic load [16,21,22,23]. The myocardium does this, due to its unique compensatory mechanism of adaptive Mt biogenesis. This can be appreciated by observing the marked increased expansion in the number of intermyofibrillar (IMF) Mt. However, if these increased stressors are sustained such as occurs in nutrient excess, the Mt will also develop impaired mitophagy as observed in obesity, IR, MetS, impaired glucose tolerance, and T2DM. Uncoupling of mitophagy from biogenesis due to impaired mitophagy and accumulation of aMt that occurs in obesity, IR, and T2DM is intriguing and not totally understood. However, there is ongoing research to figure out why impaired mitophagy is associated with Mt biogenesis. Further, this disequilibrium (Mt uncoupling) between impaired mitophagy and Mt biogenesis results in cardiomyocyte dysfunction, abnormal remodeling with fibrosis and stiffening of the heart, and even myocyte death due to apoptosis [24]. The following TEM images allow one to view myocardial Mt biogenesis and aMt accumulation due to impaired mitophagy in the female DIO-Western (Figure 6) [25], obese, female diabetic *db/db* (Figure 7) [26], and the IR, impaired glucose tolerance, hypertensive, lean, male Ren2 models (Figure 8) [27].

While the images in Figure 6 were of the 20-week-old female DIO Western models [25], similar Mt remodeling in the younger 16-week-old female diabetic *db/db* mice were noted to display increased biogenesis and impaired mitophagy with the accumulation of aMt (Figure 7) [26].

Mt fusion and fission (mitochondrial dynamics), biogenesis, and mitophagy are all important components of the Mt quality control system in the myocardium. However, the co-occurrence of both increased Mt biogenesis and importantly impaired mitophagy with aMt accumulation will result in cardiac contractile dysfunction and decreased cardiac output. The co-occurrence of impaired mitophagy will allow the leaky aMt to continuously leak an excess of superoxide—mtROS from the dysfunctional ETC into the cytosol of the cardiomyocyte. Mitophagy plays a protective role in diabetic cardiomyopathy, principally through the clearance of abnormal—dysfunctional aMt. Importantly, impaired mitophagy will be detrimental to myocardial contractility. It is important to note here that treatment with empagliflozin (a sodium glucose transporter-2 inhibitor) improved the Mt expansion and aMt in the diabetic *db/db* models with 5 weeks of treatment that were associated with improvement of diastolic dysfunction (Figure 7D, insert d) [26].

A close relationship exists between both the structural and functional relationship between Mt and the endoplasmic reticulum. This ultrastructural relationship to demonstrate the tight structural arrangement between the myocardial ER and aMt ultrastructure was identified in male Ren2 hypertensive, insulin-resistant models with impaired glucose tolerance (Figure 8) [27].

The ER and Mt are known to interact dynamically, structurally, physiologically, and functionally [28,29,30,31]. Further, this arrangement of close proximity and touching of the Mt and ER is known as the mitochondria-associated ER membrane(s) or (MAMs) [30,31]. Importantly, one of the most critical aspects of this interaction is calcium signaling and ROS exchange between these two organelles in addition to the supply of ATP from the Mt to the ER. This crosstalk between the membranes of the aMt and the ER could result in an increased transfer of both calcium and ROS. The interaction and crosstalk between these two organelles could result in ER stress that could be associated with the misfolding of proteins by the ER and further in combination with ROS leakage from the aMt could lead not only to cellular dysfunction with loss of function but also cell death via apoptosis [30,31]. The Mt-ER close interaction allows for 3 major functions, which include (1) calcium buffering; (2) Mt fission through Drp-1; (3) phospholipid transfer [31]. Importantly, it is also known that low levels of mitofusin 2 have been shown to have a positive correlation with obesity and T2DM [31]. However, if the Mt have undergone remodeling to an aberrant phenotype (depicted in Figure 8) there will develop an excess of extramitochondrial calcium accumulation. Normal Mt are known to act as a sink for calcium and acts as a calcium buffering mechanism. The release of Mt calcium content from aMt may induce cardiomyocyte apoptosis.

## 4. Mitochondria Remodeling in the Brain

**Figure 9:**
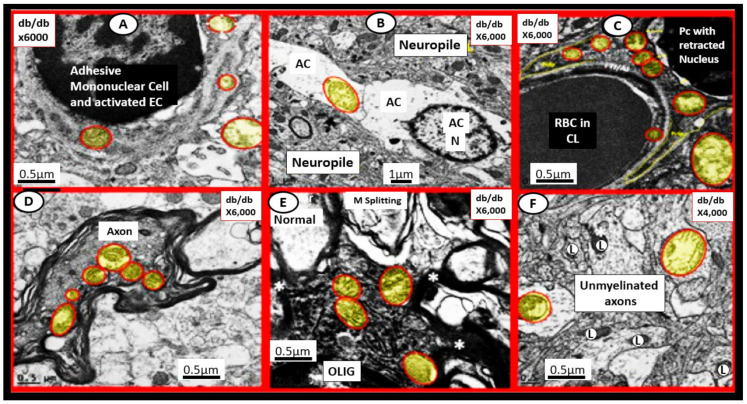
Aberrant mitochondria (aMt) are present in multiple cell types in the brain in the female 20-week-old diabetic *db/db* mouse. In contrast to the electron-dense Mt found in control healthy Mt, the aMt (pseudo-colored yellow and outlined in red) are hyperlucent with loss of electron-dense matrix and loss of cristae that are found in the brain endothelial cells (EC) (**panel** **A**), astrocyte (AC) (**panel** **B**), pericytes (Pc) and their foot processes (**panel** **C**), myelinated neuronal axons (**panel** **D**) and unmyelinated axons (**panel** **F**), and oligodendrocyte (OLIG) (**panel** **E**). These aMt are in addition to the aMt found in reactive-activated microglia in next Figure 10. Note that there is no control image in this figure; however, the control image in **panel** **A** in the next Figure 10 may be utilized as a control for this image since they were from the same experimental study. These modified images are provided by CC 4.0 [32,33,34,35,36]. Magnifications vary (upper panels) and scale bars (bottom panels) are included in each panel. Asterisk = Myelin (M); L = lysosome; RBC = red blood cell.

Brain mitochondria are essential to regulate neuronal synaptic transmission with high energetic requirements. Therefore, the remodeling of the normal Mt to an aMt phenotype could result in a loss of these normal functions and result in neuronal dysfunction in T2DM due to impaired mitochondrial bioenergetics and brain hypometabolism [32,33,34,35,36,37]. Mitochondrial dysfunction and damage are implicated in the pathogenesis of multiple neurological diseases. Virtually, all of the cells within the brain frontal cortex in layer III of the grey matter in the obese diabetic, female 20-week-old *db/db* models demonstrate aMt (Figure 9) [32,33,34,35,36].

**Figure 10:**
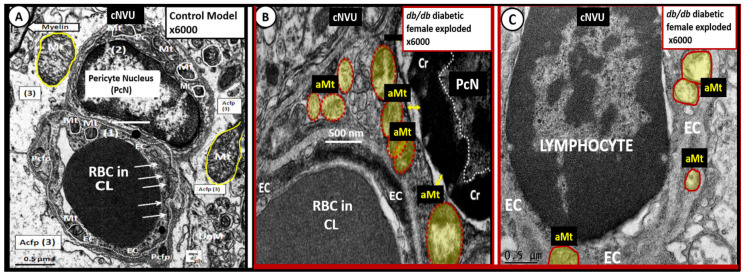
The mural pericyte (Pc) and endothelial cell (EC) of the capillary neurovascular unit (NVU) develop remodeled aberrant mitochondrial (aMt) in the 20-week-old female diabetic *db/db* models. **Panel A** demonstrates the normal electron-dense mitochondria (Mt) in the Pc and EC (outlined with white lines) in the cytoplasm. Additionally, note the arrows pointing to the tight and adherens junctions and normal electron-dense Mt in the astrocyte foot processes (outlined in yellow). **Panel B** depicts the aMt (pseudo-colored yellow outlined in red) in the Pc cytoplasm as compared to the normal Mt in **panel A**. **Panel C** depicts the aMt in the EC (pseudo-colored yellow outlined in red) as compared to the normal Mt in **panel A**. Note the excessive chromatin (Cr) condensation in the Pc nucleus. **Panels B** and **C** are exploded cropped images and these images were observed during the studies in references [32,33,34,35,36] provided by CC 4.0. Original magnification ×6000 exploded with intact scale bars of 500 nm (**panel B**) and 0.5 μm (**panels A** and **C**). Acfp = astrocyte foot process; CL = capillary lumen; Pcfp = pericyte foot processes; PcN = pericyte nucleus; RBC = red blood cell(s).

The mural cells of the neurovascular unit including the pericyte and endothelial cells (ECs) undergo remodeling of Mt to become aMt in the diabetic *db/db* models (Figure 10).

**Figure 11:**
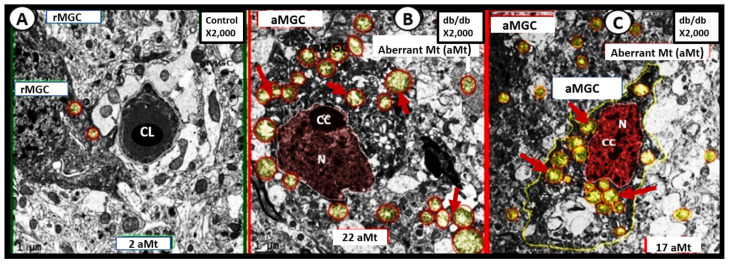
Aberrant mitochondria (aMt) in activated microglia cell(s) (MGCs) in the 20-week-old female diabetic *db/db* models. **Panel A** demonstrates a ramified MGC (rMGC) interacting with the neurovascular unit (NVU) in the control model. **Panel B** depicts an activated MGC (aMGC) with numerous aMt (pseudo-colored yellow with encircling red lines) in the diabetic *db/db* model. Note chromatin condensation (CC) within the nucleus (pseudo-colored red) (N). **Panel C** further depicts aMt and note that there is chromatin condensation within the pseudo-colored red nucleus (CC) in the female diabetic *db/db* model (**panels B** and **C**). Images provided by CC 4.0 [33,36]. CL = capillary lumen.

Additionally, reactive-activated microglia cell(s) (aMGCs) were also found to undergo marked Mt remodeling to form aMt in these same models discussed previously (Figure 11) [33,36].

These aMGCs depicted not only Mt remodeling to develop aMt but also these same MGCs underwent concurrent remodeling from a ramified state to a reactive-activated state, which encircled and damaged the neurovascular unit. This resulted in blood-brain barrier disruption in brain endothelial cells (BEC) and remodeling of the nucleus to form nuclear chromatin condensation. The neuroglia cells including the aMGCs, astrocytes (ACs), and oligodendrocytes demonstrated aMt remodeling in the above models, for those who are interested in viewing both the ramified and activated MGCs and their aMt a video has been created to demonstrate these remodeling changes in an en bloc focused ion beam scanning electron microscopy FIB/SEM video in the supplement [33].

**Figure 12:**
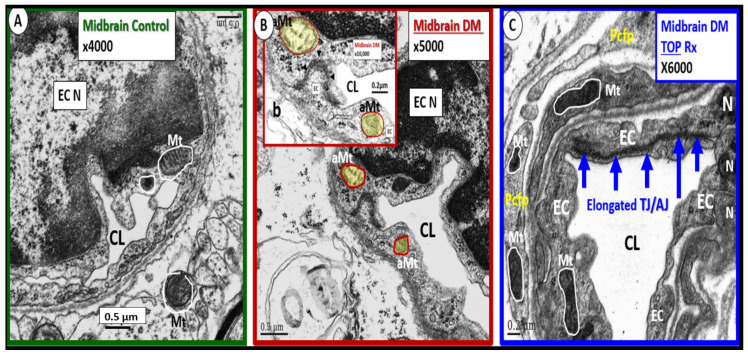
Mitochondria remodeling to an aberrant phenotype (aMt) in the male CD-1 mouse midbrain neurovascular unit brain endothelial cell(s) (EC) with streptozotocin-induced diabetes that was ameliorated with topiramate treatment. **Panel A** demonstrates the normal electron-dense mitochondria (Mt) in the endothelial cell (EC) cytoplasm (outlined in white lines) in the control model of the neurovascular unit (NVU) in the midbrain. Additionally, note the electron-dense Mt in the adjacent astrocyte of the NVU. **Panel B** depicts the aberrant mitochondria (aMt) in the EC NVU that are hyperlucent with loss of electron-dense Mt matrix and cristae (pseudo-colored yellow outlined in red). Insert (**b**) depicts this image at higher magnification. **Panel C** illustrates the electron-dense Mt (outlined in white lines) within the EC cytoplasm and the adjacent pericyte foot process (Pcfp) of the NVU. Additionally, note the elongated tight and adherens junction (TJ/AJ) of the EC (blue arrows) responsible for the blood-brain barrier of the NVU. Further, note that topiramate (TOP) treatment ameliorated the aMt in the diabetic midbrain in panel C. These modified images are from the experiment with permission by CC 4.0 in reference [38]. Magnifications vary and are located on the upper right-hand side and scale bars are located on the bottom left-hand side of each image. CL= capillary lumen; N = nucleus.

Importantly, there is a specific Mt carbonic anhydrase inhibitor that has been observed to have an effect on protecting healthy Mt from undergoing aMt changes named topiramate. Topiramate (approved for the treatment of seizures) has been shown to decrease ROS and oxidative stress in the brain by the specific inhibition of the Mt carbonic anhydrase in streptozotocin-induced diabetes [38,39,40,41,42]. In the male CD-1 streptozotocin-induced diabetic model, the brain had increased permeability to ^14^C-sucrose due to the attenuation and/or loss of the BBB via the attenuation or loss of EC tight and adherens junctions. Additionally, this model had an attenuation and/or loss of the supportive pericytes of the neurovascular unit due to increased oxidative stress at 16-weeks post-induction of diabetes. This model is known to have increased oxidative stress and increased permeability in the midbrain. In this same experiment, observations of aMt were present in the EC cytoplasm that were prevented with topiramate (50 mg/kg body weight) (Figure 12) [38].

These brain observational images strongly support the central finding of aMt in multiple brain cells in obesity, IR, and T2DM in the diabetic *db/db* models and streptozotocin-induced diabetes in the brain. Impaired brain insulin signaling due to IR is related to an increased risk of late-onset Alzheimer’s disease (LOAD) [36]. There are at least five major intersecting links to consider when discussing the relationship of T2DM and the increased risk of LOAD: (1) Aging; (2) Metabolic (hyperglycemia and advanced glycation end products (AGE) and its receptor RAGE resulting in AGE/RAGE interactions and hyperinsulinemia—IR (a linking linchpin); (3) Oxidative-redox stress (ROS, RONS, RSS) resulting in a RONSS interactome as discussed in this review of the leaky aMt; (4) Inflammation (peripheral macrophages and importantly the central brain microglia); (5) Vascular (macrovascular with accelerated atherosclerosis-vascular stiffening and microvascular NVU/neuroglial remodeling) with subsequent impaired cerebral blood flow. Peripheral IR and brain IR contribute to impaired mitophagy and disrupts the lysosomal degradation pathway [43].

**Figure 13:**
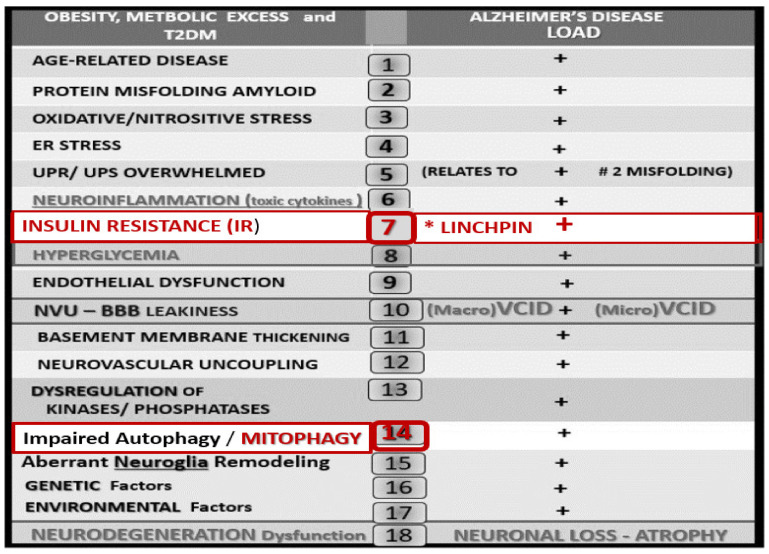
There are multiple intersecting risks between obesity, metabolic syndrome (MetS), insulin resistance (IR), type 2 diabetes (T2DM), and late-onset Alzheimer’s disease (LOAD). This figure depicts at least 18 intersecting risks or links between these two disparate chronic age-related diseases. Importantly, these intersecting risks may contribute to the increased risk of LOAD in those individuals with obesity, MetS, IR, and T2DM. Additionally, advanced glycation end products (AGE) and its receptor, the receptor for advanced glycation end products (RAGE) are important to number 8 hyperglycemia in generating reactive oxygen species and AGE could also complete with RAGE for Aβ degradation. Note that IR (number seven with asterisk) is highlighted in red as it may be considered to be the linchpin between T2DM and LOAD and further emphasizes its central role and location of IR in the MetS in previous Figure 4. Additionally, note that impaired autophagy/mitophagy (number 14) is also highlighted in red due to its role in the accumulation of aberrant mitochondria. Modified with permission by CC 4.0 [36]. Aβ = amyloid beta; BBB = blood-brain barrier; ER = endoplasmic reticulum; NVU = neurovascular unit; UPR/UPS = unfolded protein response/unfolded protein response—endoplasmic reticulum stress.

Since T2DM and LOAD are both age-related and chronic diseases, it is not unusual for them to co-occur along with cerebrocardiovascular disease in aging global societies. Both have multifactorial causations and risks with IR as a linking linchpin between these two disparate diseases (Figure 4 and Figure 13).

A great deal still remains unknown; however, a wonderful discussion regarding what is and what is not known regarding brain insulin resistance (BIR) can be found and is strongly suggested [43,44,45]. Indeed, peripheral IR is a definite core feature of T2DM that is rapidly emerging as a core feature in LOAD as in BIR, which may be defined as the failure of brain cells to respond to insulin [45,46]. Additionally, the combination of T2DM and LOAD suggest that the two disparate diseases may be synergistic when they co-occur and demonstrate the greatest decrease in insulin signaling proteins [47].

Glutamate (an excitatory neurotransmitter), plays a major role in determining certain neurological disorders including LOAD and Parkinson’s disease (PD). Glutamate neurotoxicity (GNT) is characterized by increasing damage of cellular components and organelles, including mitochondria. Since the aMt phenotype is present in BIR and T2DM, the Mt will be become increasingly dysfunctional and damaged due to GNT [48].

Indeed, aMt dysfunction, neuronal dysfunction, and damage are key features in T2DM, LOAD, and PD that can lead to synergistic neuronal toxicity and death [49]. Importantly, bacterial and viral infections are of current interest since the SARS-CoV-2 virus can lead to neuroglial activation and eventually lead to neurodegenerative progressive syndromes that have been proposed to be an additional trigger for LOAD and PD [50]. This may be very pertinent since we are all still in the midst of the coronavirus disease-19 (COVID-19) pandemic that may place some survivors at an increased risk for developing neurodegenerative diseases such as LOAD and PD over the future coming years. This may be especially true in vulnerable individuals with obesity, MetS, IR, and T2DM with dysfunctional and damaged BECs, aMt, and neuroglia activation with the aberrant remodeling of the NVU with dysfunctional and damaged BECs and endothelial glycocalyx [50,51].

**Figure 14:**
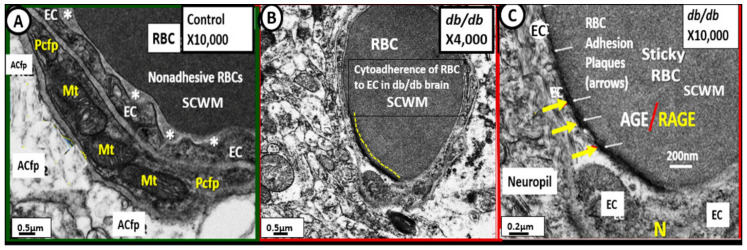
Cytoadherence of red blood cell(s) (RBCs) to the neurovascular unit (NVU) brain endothelial cells (BECs) in subcortical white matter (SCWM) regions in the obese, insulin resistant diabetic *db/db* mice. **Panel A** demonstrates the relationship between the NVUs RBC and the BEC. Note that while the RBC comes in close proximity to the BEC, it does not adhere to it. Asterisks denote a definite space between the RBC and BEC. **Panel B** depicts a low magnification image of the NVU and note that the RBC in this capillary lumen appears to be adherent to the BEC (yellow dashed line). **Panel C** depicts a definite highly electron-dense adhesion plaque between the capillary RBC and the endothelium (arrows) of the neurovascular unit. There is known to be an increase in RBC HbA1c and advanced glycation end-products (AGE) that may locate to the surface of RBCs and this could bind to the increase in the receptors of the advanced glycation end-products RAGE known to be increased in the BECs of diabetes. This AGE/RAGE interaction helps to better understand this attraction between the RBC and BEC and additionally helps to understand the formation of the electron-dense adhesion plaques observed. Additionally, the BEC glycocalyx is known to be damaged and attenuated in the diabetic *db/db* pulmonary capillaries and the diabetic BTBR *ob/ob* preclinical mouse models that would also support RBC cytoadhesion (Section 5.1). Incidentally, there were similar RBC cytoadhesions in left ventricle capillaries of the myocardium in female *db/db* mice at 20-weeks of age in this same model. Images modified with permission by CC 4.0 [32,36]. Magnification ×10,000; scale bar = 0.5 μm (**panel A**), ×4000; scale bar= 0.5 μm (**panel B**), ×10,000; scale bar = 0.2 μm (**panel C**). ACfp = astrocyte foot processes; Mt = mitochondria; Pcfp = pericyte foot processes.

Importantly, cytoadherence of red blood cells to BECs of the NVU was found to be present in the subcortical white matter (SCWM) regions of the diabetic *db/db* models (Figure 14) [32,36].

### 4.1. Brain Endothelial Cell Glycocalyx (ecGCx) of the Neurovascular Unit (NVU)

**Figure 15:**
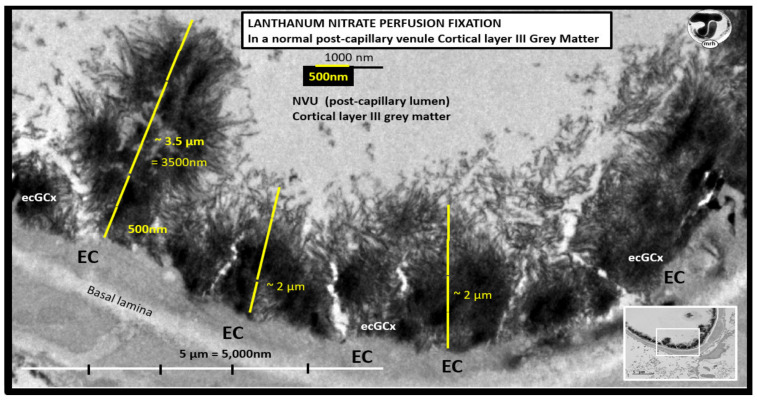
Exploded transmission electron microscope (TEM) image of a perfusion fixed control. lanthanum nitrate-stained post-capillary venule in cortical layer III of the frontal grey matter regions in a CD1 mouse model at seven-weeks of age. Note the marked electron-dense endothelial glycocalyx (ecGCx) on the luminal-apical brain endothelial cell (BEC). In this healthy control model, the ecGCx varies from ~2 to 3.5 μm in thickness. Note how the apical fronds of the ecGCx can readily interact with proteins of the circulating plasma. This staining technique has now been found to be both reliable and reproducible by author and others [52,57,58]. Note the original image in the lower-right insert with scale bar = 5 μm. Scale bar = 500 nm utilized to measure the ecGCx thickness in the exploded image.

**Figure 16:**
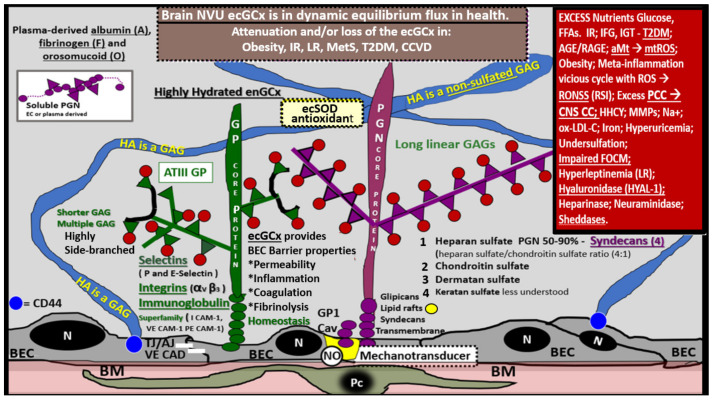
Brain endothelial cell glycocalyx (ecGCx) of the neurovascular unit (NVU). This illustration depicts the components of the ecGCx: a unique extracellular matrix. The ecGCx is composed of two classes of proteins that are mostly anchored (proteoglycan(s) (PGN) (purple), glycoprotein(s) (GP) (green)) and of hyaluronic acid (HA) hyaluronan (an exceedingly long polymer of disaccharides considered to be a glycosaminoglycan (GAG)) (blue). HA may be either unattached (free-floating), attached to CD44 brain endothelial cell(s) (BEC) plasma membrane, or form HA-HA complexes. Non-sulfated HAs not anchored to the BECs may reversibly interact at the lumen with plasma-derived albumin, fibrinogen, and soluble PGNs. The PGNs and GPs side chains consist of glycosaminoglycans (GAGs) covalently bound to core proteins and are highly sulfated (red dots). The two primary PGNs are syndecans and glypicans (purple). The glycoproteins (green) consist primarily of selectins (P and E), integrins (alpha v and beta 3) and immunoglobulin superfamily of ICAM-1, VE-CAM, and PE CAM. Caveolae are invaginations of lipid rafts in the endothelial plasma membrane and contain CD44, which anchors hyaluronan to the BEC plasma membrane, and glycosylphosphatidylinositol (GPI), which anchors glypican-1. The GPI/glypican-1 interaction is thought to be responsible for mechanotransduction in order to activate endothelial nitric oxide synthase (eNOS) to produce bioavailable nitric oxide (NO) via the calcium-calmodulin-dependent Caveolin-1 (Cav-1) protein. Importantly, note the red box (upper right), which contains a brief summary of factors that may be responsible for the attenuation and/or loss (shedding) of the ecGCx. Image modified with permission by CC 4.0 [52]. AGE/RAGE = advanced glycation end-products and its receptor; aMt = aberrant mitochondria; ATIII = antithrombin III; CD44 = cluster of differentiation 44; CNS CC = central nervous system cytokines/chemokines; ecSOD = extracellular superoxide dismutase; FFAs = saturated free fatty acids; FOCM = folate-mediated one-carbon metabolism; HHCY = hyperhomocysteinemia; IFG = impaired fasting glucose; IGT = impaired glucose tolerance; IR = insulin resistance; LR = leptin resistance; ox-LDL-C = oxidized low-density lipoprotein-cholesterol; MMPs = matrix metalloproteinases; mtROS = mitochondrial reactive oxygen species; Na+ = sodium; N = nucleus; PCC = peripheral cytokine/chemokines; RAGE = receptor for AGE; RONSS = reactive oxygen, nitrogen, sulfur species; ROS = reactive oxygen species; RSI = reactive species interactome; TJ/AJ = tight junctions/adherens junctions; T2DM = type 2 diabetes mellitus; VE CAD = vascular endothelial cadherin.

**Figure 17:**
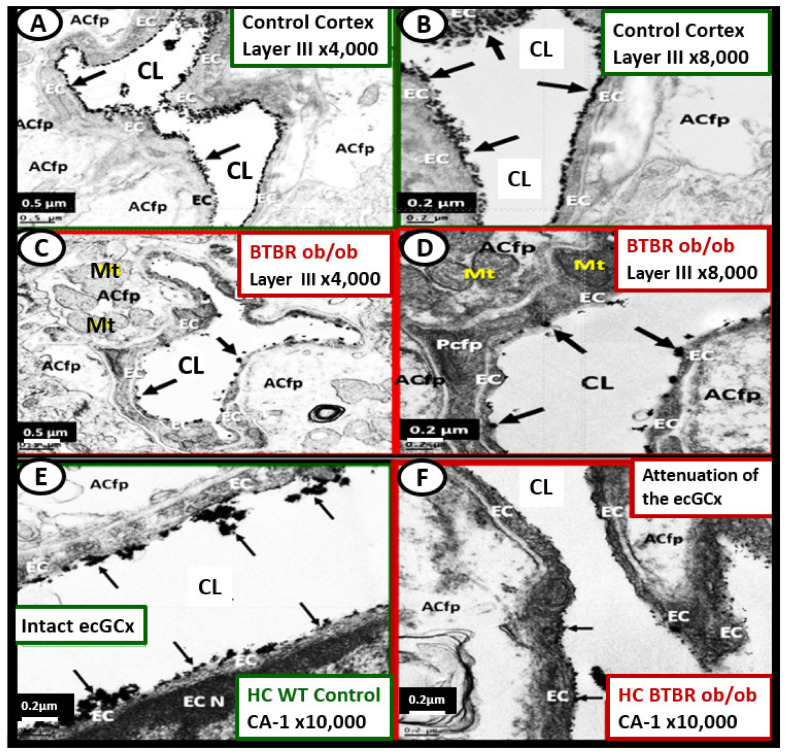
Lanthanum nitrate (LAN) staining of the neurovascular unit (NVU) endothelial glycocalyx (ecGCx) brain endothelial cells (BECs) in the female, obese, insulin resistant, diabetic BTBR ob/ob preclinical mouse models at 20-weeks of age reveal an attenuation and/or loss of the ecGCx as compared to control models. **Panels A and B** demonstrate the marked electron-dense staining of the ecGCx (arrows) on the luminal surface of BECs with LAN perfusion fixation. **Panels C** and **D** depict a marked attenuation of the ecGCx and even in some area it appears to be completely lost. Note that the ecGCx appears to clump and that it is discontinuous as compared to the continuous coverage in control models (**panels A** and **B**). **Panel E** demonstrates the decoration of the BECs by the ecGCx in the hippocampal CA-1 regions in control models. **Panel F** depicts an almost complete loss of the ecGCx on the surface layer of BECs as compared to the control (panel E). Here it is important to note that there were no mitochondria abnormalities observed in the BTBR *ob/ob* models as compared to images that were present in the *db/db* models previously discussed. Overall, we found the ultrastructural remodeling changes to be less in the BTBR *ob/ob* models as compared to the *db/db* in regard to NVU pericytes, ACs, and BBB tight junction abnormalities including less brain atrophy as compared to the *db/db* models. This may suggest that ecGCx may be an early remodeling change in the diabetic BTBR *ob/ob*. Images modified and provided by CC 4.0 [52]. Magnification ×4000; scale bar = 0.5 μm (**panels A** and **B**); Magnification x8000; scale bar = 0.2 μm (**panels B** and **D**); Magnification ×10,000; scale bar = 0.2 μm (**panels E** and **F**). ACfp = astrocyte foot processes; CA-1 = Cornu Ammonis-1; CL = capillary lumen; HC = hippocampus; Mt = mitochondria; Pcfp = pericyte foot processes.

The ecGCx is vasculoprotective and acts as the first barrier of the tripartite BBB of the NVU, which consists of: (1) ecGCx; (2) BEC; (3) abluminal basement membrane (BM), pericytes and pericyte foot processes within the BM, and astrocyte foot processes [52]. The ecGCx also covers the stromal fenestrated capillaries and the epithelial ependymal glycocalyx of the blood-cerebrospinal fluid barrier [52]. An intact ecGCx is important for the vascular integrity of arteries, arterioles, capillaries, and post-capillary venules. It is a gel-like, sugar-protein protective surface layer coating of the luminal BEC of the NVU. This protective surface coating delimits the blood and its constituents (erythrocytes, leukocytes, and thrombocytes-platelets). The ecGCx is primarily synthesized by the BECs with contributions by plasma albumin, orosomucoids, fibrinogen, glycoproteins, and glycolipids [50,51,52,53,54,55,56]. The ecGCx is anchored to the BEC luminal plasma membranes by highly sulfated proteoglycans (syndecans and glycipans), glycoproteins (including selectins such as various cellular adhesion molecules and integrins), and non-sulfated hyaluronan (a glycosaminoglycan) via BEC cluster of differentiation 44 (CD44). Hyaluronan (HA) may also be free-floating (unbound), attached to the assembly proteins such as the BEC hyaluronan synthases, or Form HA-HA stable complexes. The ecGCx is also anchored via the proteoglycan (glypican) to the caveolae and this plays a key role in mechanotransduction of BEC luminal fluid shear stress-induced synthesis of essential nitric oxide (NO) via glypican caveolae interactions located within the BEC lipid rafts (Figure 15, Figure 16 and Figure 17) [52,53]. The ecGCx has a net negative charge largely due to the sulfation of glycosaminoglycan side chains which allow for strong electrostatic binding to the polyvalent cation lanthanum nitrate (La(3+) nitrate) (LAN). This allows the ecGCx to be identified with TEM staining in perfusion fixed animal models (Figure 15) [52,57].

The diffuse electron density of the ecGCx does not allow one to identify any substructures within this surface layer of the EC, and therefore, an illustration has been generated to demonstrate its contents (Figure 16).

The ecGCx serves as the first luminal barrier of the NVU and lanthanum nitrate (LAN) staining has been utilized to define the ecGCx by our laboratory and others [52,57,58]. LAN staining allows the ecGCx to be visualized with TEM studies due to the strong electrostatic attraction between the highly positive charge of LAN to the negatively charged ecGCx. We have previously utilized LAN staining in the diabetic BTBR *ob/ob* models and found it to be both reliable and reproducible [52], while others have utilized LAN staining in the diabetic *db/db* models in the pulmonary system, which demonstrated an attenuation of the ecGCx in the diabetic *db/db* preclinical models of the lung [58]. LAN staining revealed that the ecGCx was markedly attenuated and even lost in the obese, insulin-resistant, diabetic BTBR *ob/ob* models in the frontal cortical layer III and CA-1 regions of the hippocampus (Figure 17) [52].

**Figure 18:**
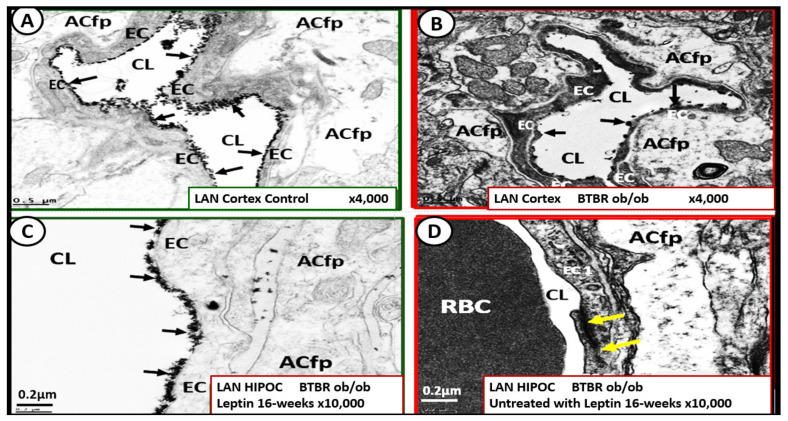
Leptin replacement in the obese diabetic BTBR *ob/ob* protects the brain endothelial cell glycocalyx (ecGCx) in cortical layer III and hippocampus. **Panel A** demonstrates the continuous decoration of the ecGCx with lanthanum nitrate (LAN) staining in the heterozygous non-diabetic control model cortical layer III (arrows). **Panel B** depicts the marked attenuation and/or loss of the ecGCx in the obese diabetic BTBR *ob/ob* model cortical layer III and note when the ecGCx was present it was clumped and discontinuous (arrows). **Panel C** also demonstrates the continuous decoration of the ecGCx in hippocampus CA-1 regions of the BTBR *ob/ob* models that were treated with intraperitoneal leptin for 16-weeks and stained with LAN (arrows) and the ecGCx is comparable to the control model in **panel A** and that the ecGCx is continuous. **Panel D** depicts the complete loss of the ecGCx by LAN staining in the hippocampus CA-1 regions of the BTBR ob/ob and note that the tight and adherens junction (TJ/AJ) remain intact (yellow arrows). The loss of the first barrier of the tripartite BBB may result in increased permeability. Images provided by CC 4.0 [52]. Magnification ×4000; scale bar = 0.5 μm in **panels A** and **B**. Magnification ×10,000; scale bar = 0.2 µm in **panels C** and **D**. ACfp = astrocyte foot process; Cl = capillary lumen; BEC = brain endothelial cell; HIP and HC = hippocampus CA-1 regions.

Interestingly, when the BTBR *ob/ob* models were treated for 16-weeks prior to sacrifice with leptin (15 μg/day via implanted intraperitoneal pump), it was observed that restoring leptin protected the ecGCx from being attenuated and/or lost (Figure 18) [52].

**Figure 19:**
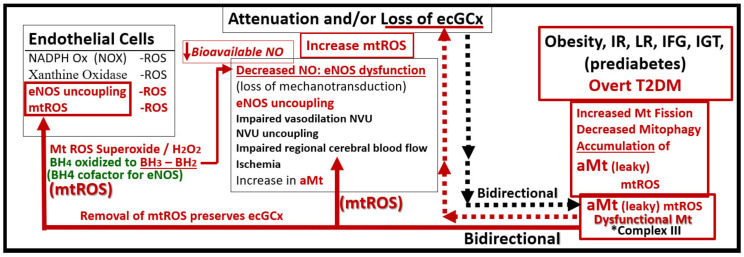
Emerging evidence may suggest a possibility for a bidirectional relationship between aberrant Mt (aMt) and the endothelial glycocalyx (ecGCx) dysfunction and/or loss in endothelial cells, including brain endothelial cell(s) (BECs) and possibly other organs in obesity, insulin resistance (IR), leptin resistance (LR), metabolic syndrome (MetS), and type 2 diabetes mellitus (T2DM). This figure illustrates that there may be a bidirectional relationship between aMt and dysfunction, attenuation, and/or loss (shedding) of the brain BECs ecGCx. Note that this illustration establishes the role of oxidant stress—reactive oxygen species (ROS) in BECs and specifically mitochondrial ROS (mtROS) (left-hand box). Further, it establishes the important role of the dysfunctional attenuated and/or loss of the ecGCx in the brain (center box). Next, it establishes that obesity, IR, LR, impaired fasting glucose (IFG), impaired glucose tolerance (IGT), and overt T2DM are related to increased Mt fission, decreased mitophagy, and the accumulation of leaky aMt that leak mtROS (right-hand box). Further, this image depicts that these leaky aMt may be responsible for the attenuation and/or loss of the ecGCx (red-dashed arrows) and that, in turn, may result in the loss of the ecGCx that may contribute to an increase in aMt (black-dashed arrows). Importantly, mtROS superoxide or H_2_O_2_ could oxidize the essential tetrahydrobiopterin (BH4) cofactor that is absolutely essential for the eNOS enzyme to produce nitric oxide (NO) and result in eNOS uncoupling, which would ultimately result in decreased bioavailable NO. This bidirectional interaction could result in a vicious cycle, which could be interrupted by preventing the accumulation of aMt (improved mitophagy) or preventing the dysfunction, attenuation, and/or loss (shedding) of the ecGCx [38,59,60,61,62,63,64,65,66,67,68]. The asterisk signifies emphasis of complex III.

If the NVU ecGCx becomes dysfunctional, attenuated, and/or lost via shedding as in the obese, insulin-resistant diabetic BTBR *ob/ob* model [52], then there would be a decrease in bioavailable NO to signal pericytes in the capillary NVU and NVU uncoupling would develop due to the loss of regional mechanotransduction at these BEC regions. This would result in regional decreased cerebral blood flow and regional ischemia. If ecGCx loss is an early event, as suspected in the BTBR ob/ob models, this would add to the oxidative stress of the BECs and possibly contribute to an even greater increase in mtROS as well as NADPH oxidase-derived ROS. An intact glycocalyx and Mt of BECs are of utmost importance in maintaining the NVU BBB homeostasis. Damage to the glycocalyx in T2DM can have multiple pathophysiological consequences, which include: (1) increased vascular permeability; (2) edema formation; (3) increased adhesion of circulating inflammatory cells to the endothelium; (4) accelerated inflammatory processes; (5) activation of the coagulation cascade; (6) platelet aggregation [50,51,52,53,54,55,56]. Excess nutrients (increased glucose and fatty acids) in obesity, MetS, IR, T2DM, and the associated glucotoxicity will result in leaky aMt phenotypes and an increase in mtROS. In turn, this will lead to dysfunction, attenuation, and/or shedding of the ecGCx and a further increase of aMt and mtROS. Additionally, this increase in EC mtROS could result in contributing to even further ecGCx shedding or impairment in ecGCx regeneration. This sequence of events between aMt, mtROS, and ecGCx shedding could be bidirectional, such that a vicious cycle may ensue wherein one abnormality may lead to another. Decreasing mtROS from leaky aMt results in improved homeostasis [35,38,57,58,59,60] and likewise restoring the ecGCx also improves homeostasis and function [60,61,62,63]. ecGCx with attenuation and/or shedding in BECs may be playing a bidirectional role in the development and progression of impaired cognition and neurodegeneration. Importantly, there may exist a bidirectional role between the accumulation of aMt (due to impaired mitophagy) and the dysfunction or loss of the ecGCx in BECs (Figure 19) [32,33,34,35,36,59,60,61,62,63,64,65].

While more research is necessary to confirm this bidirectional relationship between aMt and ecGCx attenuation or loss via shedding, it is nevertheless a very intriguing association and presents an emerging opportunity to further unlock some of the mysteries associated with Mt in obesity, IR, MetS, and T2DM and possibly the associated complications of diabetic end-organ disease including the brain. Additionally, this concept may allow for future interventions to interrupt this bidirectional vicious cycle by utilizing Mt carbonic anhydrase inhibition with topiramate [38], uncoupling proteins such as UCP2 [61], and the emerging role of Mt transfer [62].

## 5. Descending Aorta Mitochondria Remodeling

It is important to discuss Mt remodeling in the descending thoracic aorta to better understand its relationship to vascular stiffening and the heart-brain-kidney (HBK) axis as discussed in Figure 3 and how this links aortic stiffening and its relationship for the progression to end-organ damage in HBK axis in obesity, IR, impaired glucose tolerance, MetS, and T2DM.

**Figure 20:**
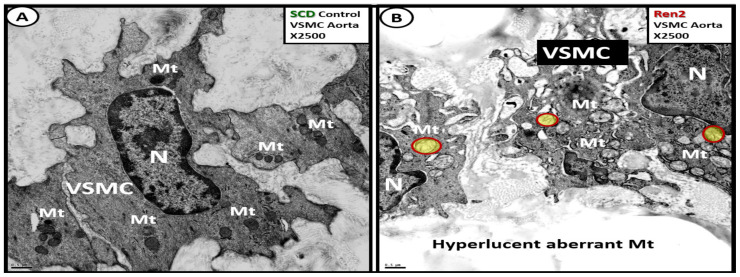
Aberrant mitochondria (aMt) were present in the 12-week-old male insulin resistant, impaired glucose tolerant, and hypertensive Ren2 rat model as compared to the Sprague Dawley controls (SDCs) in the descending thoracic aorta. **Panel A** demonstrates the normal morphology of the media vascular smooth muscle cell (VSMC) and note the normal appearance of the highly electron-dense mitochondria (Mt). **Panel B** depicts the hyperlucent aberrant Mt with loss of cristae and loss of the electron-dense Mt matrix (with some aMt pseudo-colored yellow with red outlines) in the Ren2 as compared to the SDC in **panel A**. These images are provided by permission from reference [66]. Magnification ×2500; scale bar = 0.5 μm. *N* = *nucleus*.

The vascular *tunica media*, which contains vascular smooth muscle cell(s) (VSMCs) depict aMt remodeling when there are cellular stressors such as hypertension, insulin resistance, and impaired glucose tolerance as occurs in the Ren2 descending aorta *tunica media* VSMCs (Figure 20) [66].

**Figure 21:**
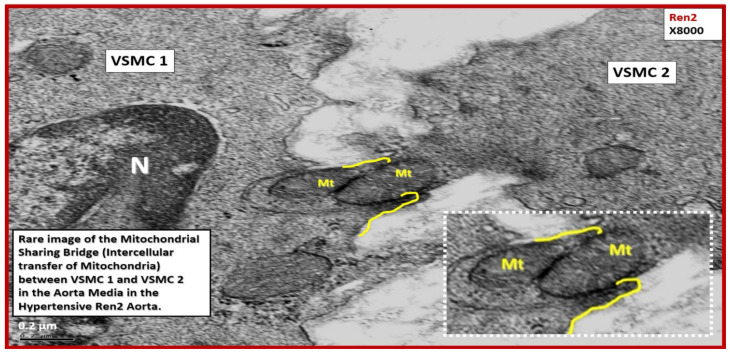
Rare image depicting intercellular mitochondria (Mt) transfer from one vascular smooth muscle cell to another in the descending aorta of the insulin resistant, impaired glucose tolerant, hypertensive 12-week-old Ren2 rat model. This image depicts the rare intercellular transfer of mitochondria between vascular smooth muscle cells (VSMC 1) and VSMC 2. Note the exploded image in the lower-right outlined by white dashed lines. It is intriguing to think that in the stressed media of the Ren2 model of hypertension that a VSMC may be capable of transferring its Mt from a healthy VSMC to one that has extensive aberrant Mt as in the previous image in Figure 20 from panel A to panel B in order to maintain VSMC homeostasis. This image is from the same experiment as in reference [66]; however, it has not been previously published. Magnification ×8000; scale bar = 0.2 μm. *N* = *nucleus*.

Cell-cell communication is a process necessary for physiological tissue homeostasis and is frequently impaired or altered in many disease states such as obesity, IR, impaired glucose tolerance, T2DM, and hypertension [66,67]. Importantly, intercellular transfer of mitochondria is rarely observed but is known to occur in certain stress states such as hypertension in the aorta [66,67]. In the stressed cells of the descending aortic *tunica media* in the Ren2 rat model, the author was able to identify this rare process between two VSMCs from an ex vivo model and indeed, Mt know no boundaries (Figure 21) [68].

**Figure 22:**
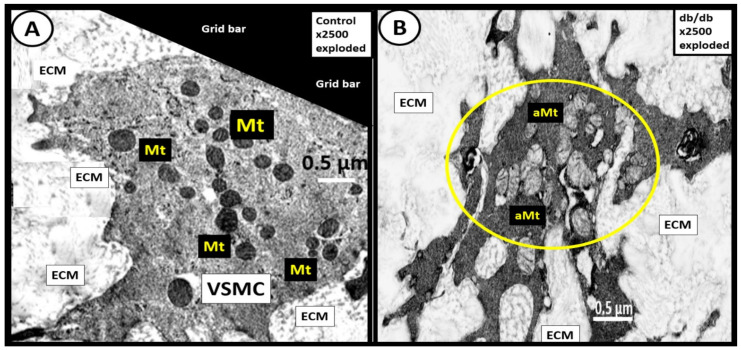
Aberrant mitochondria (aMt) in the obese, insulin resistant, diabetic *db/db* model vascular smooth muscle cell(s) (VSMCs) in the descending aorta. **Panel A** demonstrates the markedly electron-dense healthy mitochondria (Mt) in the cytoplasm of a non-proliferative VSMC. **Panel B** depicts a cluster of hyperlucent aberrant Mt (aMt) (encircled with yellow line) in the cytoplasm of a proliferative VSMC with loss of the electron-dense Mt matrix and loss of crista as compared to the control C57BLKS/J models in **panel A**. Original Magnification ×2500; intact scale bar = 0.5 μm. These images are exploded in a like manner with the scale bars intact to emphasize the normal control electron-dense Mt in **panel A** and the aMt in the *db/db* models in **panel B**. The original images for this figure are from the experiment with permission [69]. *ECM* = *extracellular matrix*.

Additionally, the obese, insulin-resistant, and diabetic *db/db* models revealed aMt in the proliferative VSMCs of the tunic media similar to the hypertensive Ren2 model [66] (Figure 22) [69].

aMt are a prominent feature in the VSMC in the hypertensive Ren2models [66]. Additionally, they are a characteristic finding in the obese, diabetic, female *db/db* models [69] and strongly suggest an important role in the remodeling of the descending thoracic aorta that results in stiffening of the thoracic aorta.

### 5.1. Perivascular Adipose Tissue (PVAT) of the Descending Thoracic Aorta in Obese, Insulin Resistant, and Diabetic db/db Model: The Tunica Adiposa of the Adventitia

**Figure 23:**
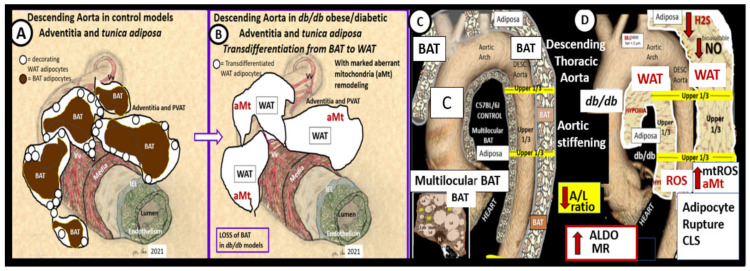
Transdifferentiation of the perivascular adipose tissue (PVAT) brown adipose tissue (BAT) of the descending thoracic aorta in control models to white adipose tissue (WAT) in the obese, insulin-resistant, diabetic *db/db* models. **Panels A** and **B** are hand drawn illustrations, while **panels B** and **D** depict images built around colorized MRI images of the aorta. **Panels A** and **C** demonstrate the normal BAT that envelops the healthy descending aorta in control models. **Panels B** and **D** depict the transdifferentiation of adipocytes from multilocular BAT in control models (**panels A** and **C**) to unilocular WAT in the obese, insulin-resistant, diabetic *db/db* models (**panels B** and **D**. This transdifferentiation from BAT to WAT of the PVAT is associated with the accumulation of aMt within the WAT adipocytes. These adipocytes become engorged and eventually rupture and develop crown-like structures associated with inflammation and loss of the PVAT protective functions, which result in aortic vascular remodeling and vascular stiffness in diabetic *db/db* models. Note that the aberrantly remodeled WAT in the obese diabetic *db/db* models (**panels B** and **D**) are associated with aMt, which are associated with increased mtROS, aldosterone (ALDO) and mineralocorticoid receptors (MR). Additionally, note the decrease in the adiponectin/leptin ratio (A/L) and the decrease in nitric oxide (NO) and hydrogen sulfide (H2S). These remodeling changes to the PVAT in the obese, diabetic *db/db* models results in the loss of the anticontractile and protective effects of the PVAT to the aortic vascular wall and promote remodeling vascular stiffening. The modified images in **panels C** and **D** are provided with permission from reference [69].

The PVAT of the descending thoracic aorta allows one to examine mitochondria remodeling in the adipose tissue of both brown adipose tissue (BAT) and white adipose tissue (WAT) depots in the *tunica adiposa* of the aortic adventitia. In healthy human individuals and preclinical rodent models, the descending thoracic aorta PVAT consists of BAT in the *tunica adiposa* in contrast to WAT in the abdominal aorta [70]. However, in obesity, insulin resistance, and T2DM as in the *db/db* model, the PVAT BAT undergoes a near complete transdifferentiation to WAT, which is dysfunctional and is associated with marked Mt remodeling to aMt and adipocytes that rupture and incite inflammation to develop crown-like structures [69]. This transdifferentiation from BAT to WAT results in the loss of the protective functions of the PVAT to the aortic vascular wall and it remodels to a stiffened vessel that is damaging to the end organs of the HBK axis capillaries with high flow and low resistance to result in end-organ dysfunction, damage, and disease. (Figure 23).

**Figure 24:**
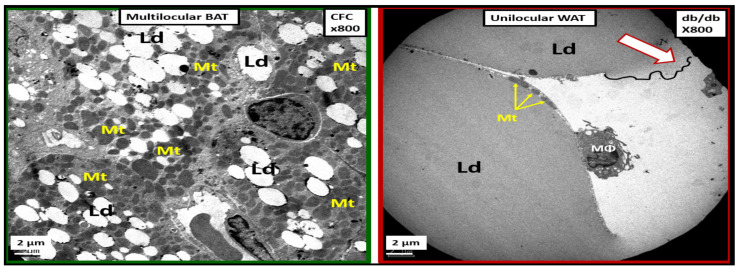
Comparison between multilocular brown adipose tissue (BAT) in control (CFC) perivascular adipose tissue (PVAT), which transdifferentiates to unilocular white adipose tissue (WAT) in the obese, insulin resistant, diabetic *db/db* model. The left-hand panel demonstrates the multilocular BAT in the CFC control models in the descending thoracic aorta PVAT with its multiple mitochondria (Mt). The right-hand panel depicts the unilocular transdifferentiated WAT with the adherence of an inflammatory macrophage (MΦ) in the diabetic *db/db* model. Note the rupture of the plasma membrane with leakage of inciting inflammatory lipids into the interstitium (open arrow) that has no Mt and also the thinned small Mt (closed arrows) in the adjacent adipocyte. This image is from the experiment in reference [69]. Ld = lipid droplet.

The transdifferentiation of PVAT from BAT to WAT (Figure 24) contributes to aortic vascular stiffening as well as remodeling damage not only in the heart but also the brain and the kidney as in the HBK axis [26,35,71]. While BAT is the predominant adipose tissue in the PVAT in control models, the *db/db* models of obesity, insulin resistance, and T2DM undergoes a remodeling transdifferentiation to WAT and aMt remodeling (Figure 24) [69].

**Figure 25:**
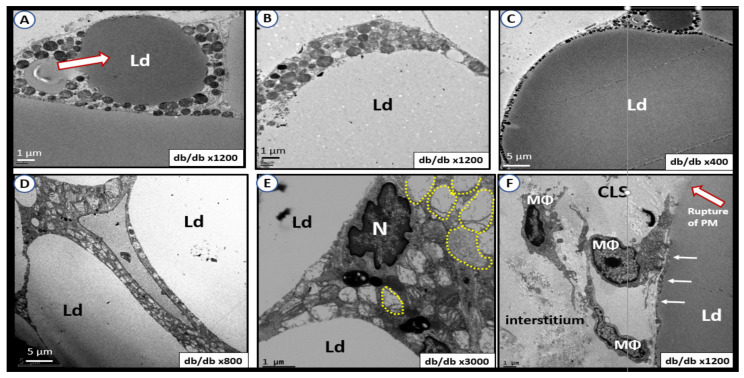
Brown adipose tissue (BAT) in controls to transdifferentiated white adipose tissue (WAT) in obese, insulin resistant, diabetic *db/db* models. **Panels A**–**F** depict the gradual and continuous deposition of lipids in the form of triglycerides into the lipid droplet(s) (Ld) of the transdifferentiated engorged WAT adipocytes in the *db/db*. Note how the mitochondria (Mt) become pushed to the outer rim of the Ld and become smaller as in the progression from **panels A**–**C**. In **panels D** and **E** note the aberrant mitochondria (aMt). **Panels D** and **E** depict the hyperlucent aberrant Mt with decreased electron density of the Mt matrix and attenuation and/or loss of crista (outlined by yellow dashed lines) in **panel E**. In **panel F** note that the plasma membrane is thinned (closed white arrows) to the point of rupture (open arrow outlined in red) with the release of toxic free fatty acids into the interstitium and the resulting recruitment of inflammatory macrophages (MΦ) to form crown-like structures (CLS). These figures are provided with permission from the experiment in reference [69]. Magnifications and Scale bars vary. *Ld* = *lipid droplet*; *N* = *nucleus*; *PM* = *plasma membrane*.

Importantly, the remodeling of the *tunica adiposa* results in the loss of the anticontractile and protective mechanisms of the normal healthy BAT in the descending thoracic aorta [69]. The transdifferentiated and remodeled WAT of the PVAT in the upper 1/3 of the descending thoracic aorta of *db/db* models demonstrates aMt and inflammatory crown-like structures (CLS) (Figure 25).

**Figure 26:**
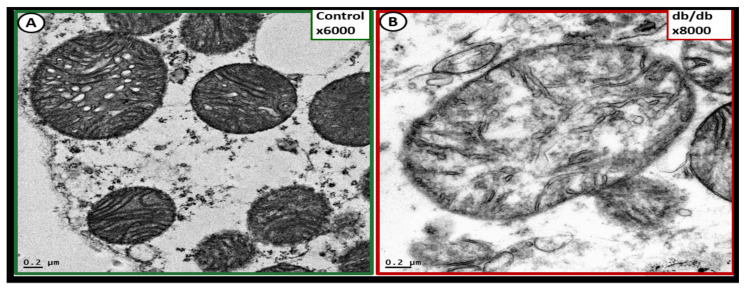
Comparison of healthy mitochondria in controls to aberrant mitochondria (aMt) in remodeled transdifferentiated white adipose tissue of the descending aorta of the obese diabetic *db/db* model. **Panel A** demonstrates the electron-dense mitochondria (Mt) in the control model and note the prominent crista and the electron-dense Mt matrix. **Panel B** depicts the hyperlucent Mt with loss of the electron-dense Mt matrix and incomplete formation and loss of crista. This aMt would be leaky of ROS—superoxide and result in a dysfunctional PVAT that is known to be associated with aortic stiffening in *db/db* models. Importantly, this highly magnified image applies to remodeling changes in the normal control Mt (**panel A**) to the aMt (**panel B**) as found in disease models in all the ultrastructure presented images. These figures are provided with permission from the experiment in reference [69]. Magnification ×6000 and ×8000; scale bar = 0.2 μm in **panels A** and **B** respectively.

aMt in the transdifferentiated WAT adipocytes may be characterized by hyperlucency with a loss of electron-dense Mt matrix and loss of crista (Figure 26) [69].

Section 5 and Section 5.1 have shared multiple TEM images depicting multiple Mt remodeling changes in the descending thoracic aorta and these changes are important to better understand the overall remodeling of the descending thoracic aorta and aortic stiffening. Aortic vascular stiffening may be considered as the nexus between the HBK axis with subsequent cellular and Mt remodeling in these end-organs that are being affected by an abnormally increased aortic pulse pressure and the damage to the tissues and capillaries that they serve [72].

## 6. Thoracic Aorta Vascular Stiffening as the Nexus between the Heart-Brain-Kidney (HBK) Axis

**Figure 27:**
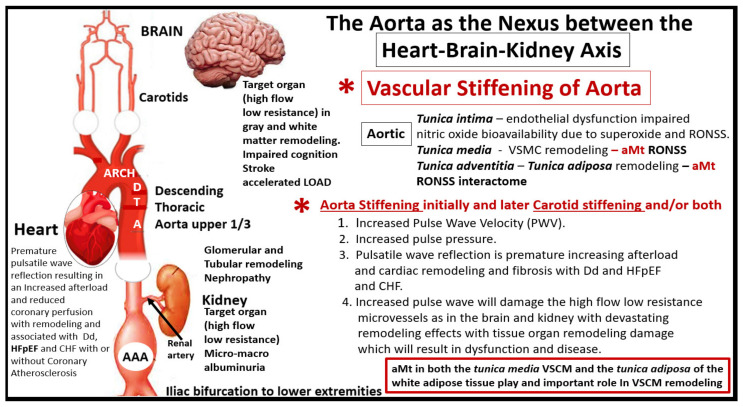
Descending thoracic aortic stiffening as the nexus between the heart-brain-kidney axis. This montage image illustrates the importance of aortic stiffening and the increased pulse wave velocity (PWV) with aberrant wave reflection to the heart and increased afterload resulting in myocardial remodeling and the increased pulsatile PWV to the target organs of high flow and low resistance microvessels especially in the capillaries of brain and kidney due to aortic stiffening with resultant damage and injury and the response to injury remodeling. Importantly, note that the aberrant mitochondria (aMt) in the *tunica media* vascular smooth muscle cells (VSMCs) and the aMt in the *tunica adiposa* of the adventitia in the descending thoracic aorta may also play an important role in thoracic aortic stiffening. A = aorta; AAA = abdominal aortic aneurysm; CHF = congestive heart failure; D = descending; Dd = diastolic dysfunction; HFpEF = heart failure with preserved ejection fraction; LOAD = late-onset Alzheimer’s disease; PWV = pulse wave velocity; ROS = reactive oxygen species; RONSS = reactive oxygen, nitrogen, sulfur species.

Vascular stiffening of the descending thoracic aorta may be considered as the nexus between the HBK axis [72]. Importantly, the combined aMt in the VSMCs in the Ren 2 of the *tunica media* [66] and the WAT of the *tunica adiposa* of the thoracic aorta adventitia [69] are very important to the development of the descending thoracic aorta stiffening (Figure 27).

A healthy elastic aorta has a phenomenal cushioning function to the high pressure exerted by systolic flow from the left ventricle of the heart, and this cushioning effect limits the arterial pulsatility and serves to protect the microvasculature in the high flow and low resistance microvessels such as occurs in the HBK axis. However, when thoracic aorta stiffening develops as in the hypertensive Ren2 and diabetic *db/db* preclinical models this protective elastic cushioning is diminished and/or lost, and these microvessels will respond to this injury in a response to injury wound healing response, which results in dysfunction and damage to the heart, brain, and kidney [26,35,71]. It is important to note that even though aortic stiffening precedes isolated systolic hypertension and is related to target organ damage in the heart, brain, and kidney that the descending thoracic aorta is also a target organ that is significantly affected by aging and other various states such as obesity, MetS, diabetes (T1 and T2DM), smoking, elevated lipids (triglycerides and low-density lipoprotein (LDL) cholesterol, and chronic kidney disease [38,73,74]. Plus, aortic stiffening has a central and important role in providing a vicious cycle of hemodynamic dysfunction that may be characterized by excessive pulsatility (increased PWV) that contributes to a HBK axis [26,35,71,72].

Indeed, aortic stiffness is a complex phenomenon that arises from multiple structural alterations in the aortic wall with impaired endothelial function, increased VSMC tone, phenotypic modulation to a proliferative phenotype of adventitial fibroblast to myofibroblasts, chronic low-grade inflammation, and the loss of the PVATs *tunica adiposa* anticontractile effect [72,73,74]. Increased aMt in VSMCs in hypertension, obesity, MetS, and T2DM suggests that increased superoxide (mtROS) along with other RONSS interactome would over time deplete the vascular wall antioxidants such as superoxide dismutases (MnSOD, CuZnSOD, EcSOD), catalase, glutathione peroxidase, paraoxonase, thioredoxin peroxidase, and heme oxygenases [73] and contribute to aortic vascular stiffening.

Superoxide released from the aMt would also instigate the RONSS interactome in the VSMCs within the *tunica media* and result in the activation of latent matrix metalloproteinases (MMPs) with subsequent collagen type I deposition as well as elastin fragmentation and remodeling. This vascular extracellular matrix remodeling would also result in aortic stiffening with increased PWV and impaired pulsatile reflection to the heart resulting in increased afterload and remodeling of the myocardium in a stress injury response and response to injury remodeling with interstitial fibrosis [74]. Concurrently, this increased pulsatile force, which also occurs in the carotid arteries would be transferred to the brain microcirculation (neurovascular units) and similarly to the renal arteries with damaging effects to the renal glomeruli and PTCs to result in both a glomerular and tubular remodeling nephropathy with early microalbuminuria and later macroalbuminuria.

Thus, the aMt that are found within the Ren2 and the diabetic *db/db* VSMCs could result in both hypertension and, importantly, aortic vascular stiffening with damage to the target organs with high flow and low resistance such as the heart, brain, and kidney [26,35,71,72,73,74,75,76].

Elevated homocysteine levels referred to as hyperhomocysteinemia (HHcy) are a biomarker of impaired FOCM [15] and are strongly and independently correlated to arterial stiffness measured by aortic pulse wave velocity [77]. Plus, it is known that T2DM is associated with impaired FOCM and HHcy including the preclinical diabetic *db/db* models [15,77,78,79,80,81,82,83]. HHcy results in increased ROS and the RONSS interactome, which would also accelerate vascular stiffening. HHcy promotes oxidant injury to vascular cells including the endothelial cells and VSMCs via ROS due to the process of Hcy autoxidation, formation of Hcy disulfides, the interaction of Hcy thiolactones, and protein homocysteinylation [15,79,80,81,84,85]. The ROS produced by HHcy then would be capable of acting synergistically with the superoxide generated from aMt to interact with nitrogen and sulfur species to form RONSS and eventually implicate the RONSS interactome [15]. The RONSS interactome within the *tunica media* VSMCs and the *tunica adiposa* of the adventitial layer would then be capable of contributing to the overall oxidative-redox stress of the aortic vascular wall to result in remodeling and provide a compelling contribution to the thoracic descending aorta stiffening that negatively affects the HBK axis [78,83,85].

## 7. aMt Remodeling in Obesity, IR, MetS, T2DM and Other Important Organs: Skeletal Muscle, Visceral White Adipose Tissue, Liver, Pancreatic Islet β-Cells, and Kidney

Mitochondria remodeling to an aMt phenotype also occurred in soleus slow-twitch skeletal muscle [86,87]. Visceral, omental WAT had similar remodeling with aMt and the development of crown-like structures as discussed in Section 5.1 of the PVAT [86]. Liver hepatocytes developed aMt [16]. Pancreatic islet beta-cells with a concurrent dilated Golgi apparatus to suggest endoplasmic reticulum stress [86,88]. Basilar kidney proximal tubule cells had a marked remodeling change to aberrant fragmented Mt with loss of elongation and formation of spherical Mt suggesting fission with concurrent disordered and chaotic basilar canalicular system [86,89,90].

## 8. Conclusions

Mt remodeling to an aMt phenotype and Mt dysfunction is a common thread that weaves across the multiple organ systems in obesity, IR, MetS, and the T2DM mosaic fabric of disease. The images presented in this narrative and ultrastructure Mt review still brings the author back to the story and the presentation shared by Michael Brownlee when he delivered his classic presentation and later published his findings entitled: “The Pathobiology of Diabetic Complications: A Unifying Mechanism” as a title for the Banting lecture in 2004 [91]. Specifically, Brownlee had a section in this manuscript regarding putting the pieces of the puzzle together. He spoke of four puzzle pieces, including (1) increased flux across the polyol pathway; (2) advanced glycation end-products (AGE); (3) hyperglycemia-induced activation of protein kinase C; (4) increased flux across the hexosamine pathway (all due to hyperglycemia).

This review has demonstrated remodeling of the healthy Mt in control models to an aberrant phenotype (aMt) in obesity, IR, and T2DM that certainly would be an important and additional piece of this puzzle. The additional ‘fifth piece’ of the puzzle, (aMt), would increase mtROS and the RONSS interactome, which could induce DNA strand breaks and activate nuclear poly (ADP-ribose) polymerase (PARP) and result in decreased glyceraldehyde 3-phosphate dehydrogenase (GAPDH) and subsequently increase each of the four previous Brownlee puzzle pieces [92].

Mt may be considered to be unifying organelles and metabolic hubs in multiple organs of obesity, IR, MetS, and T2DM. This review has shared multiple ultrastructural TEM images depicting that Mt undergo remarkable remodeling in multiple organs and cells with a common finding of aMt depicting hyperlucency, attenuation and/or loss of electron-dense Mt matrix, and attenuation and/or loss of cristae. These aMt were a constant unifying theme in multiple different organs and cells studied in this observational TEM study.

The presence of multiple complex, interacting cycles within the Mt matrix include the folate and methionine cycles of FOCM [15]. The Mt matrix FOCM transfers one-carbon units for many biochemical processes, which include (1) purine and deoxythymidine monophosphate (dTMP) biosynthesis; (2) Mt protein translation; (3) cell proliferation, protein synthesis, and Mt respiration. Therefore, impaired FOCM in T2DM [15] may contribute to impaired cell proliferation, protein synthesis, and Mt respiration in addition to an accumulation of mtDNA deletions [15,93].

In myocardial cells (Section 3) it was quite evident that there was compensatory Mt biogenesis in the intermyofibrillar regions; however, this increase in Mt due to biogenesis was also plagued with findings of multiple aMt. This accumulation of aMt strongly suggested that there was also concurrent impaired mitophagy that allowed these aMt to accumulate and leak damaging superoxide mtROS. These superoxide free radicals would then interact with nitrogen and sulfur species to generate not only ROS but also the generation of RNS and reactive sulfur species RSS to generate the RONSS and act as a RONSS interactome to enhance the overall oxidative-redox stress within myocardial cells and the multiple cells and organs that were also shared in this review. In the brain (Section 4) these aMt would not only increase mtROS and activation of the RONSS interactome but also contribute to hypometabolism that is present in both T2DM and late-onset Alzheimer’s disease (LOAD) due to impaired oxidative phosphorylation and generation of ATP [37,39,64,94,95]. Importantly, the aMt may be related to attenuation and/or loss of the ecGCx and this relation between aMt and loss of the ecGCx may be bidirectional (Section 4.1). Additionally, there was an attenuation and/or loss of subsarcolemmal, intermyofibrillar, and pericapillary Mt in skeletal muscle (Section 7); a marked remodeling of PTCs from elongated basilar Mt to spherical Mt suggesting increased fission (Section 7); aMt in descending aorta VSMC (Section 7); PVAT *tunica adiposa* adipocytes (Section 5.1); liver hepatocytes (Section 7) and pancreatic islet beta cells (Section 7). Further, there was also an impact of aMt on the cytoadhesion of RBCs to activated ECs in the capillaries of the diabetic *db/db* models in the brain (Section 4—Figure 14).

Novel emerging information has identified the presence of specific mitochondrial matrix metalloproteinases (MMP-2 and MMP-9). These proteinases-gelatinases (referred to as moonlighting proteins) would be capable of creating an increase in MOMP and the release of mtROS and cytochrome c due to nutrient excess such as hyperglycemia and increased superoxide, which adds another dimension to the formation of leaky aMt [96].

The persistent findings of aMt hyperlucency, vesiculation/vacuolization, loss of Mt matrix electron density, and cristae strongly suggest impaired mitophagy. Importantly, healthy Mt cardiolipin (a phospholipid of the inner membrane) not only plays an important role in the process of energy generation of ATP via the ETC but also in the process of preserving mitophagy. Therefore, if cardiolipin is impaired or decreased as occurs in obesity, IR, MetS, and T2DM, its functional impairment due to increased mtROS within the Mt inner membranes could impair not only Mt ETC function in producing ATP but also mitophagy [97,98,99].

Importantly, in Section 3 the ultrastructural findings of increased Mt biogenesis and impaired mitophagy were demonstrated (Figure 6, Figure 7 and Figure 8); however, the possible causes for this remodeling phenomenon were not discussed. In this regard, Palikaras et al. [24,100] have shared that there is a delicate balance wherein mitochondria biogenesis and mitophagy are coupled in order to maintain cellular homeostasis. However, uncoupling of Mt biogenesis and mitophagy may result in dyshomeostasis with impaired mitophagy, which results in the accumulation of aMt as previously discussed and depicted in different models, cells, and organs in previous Section 3. This group has been actively working with *Caenorhabditis elegans* in order to sort out these complicated interacting mechanisms and what may be done in the future to prevent Mt biogenesis and mitophagy uncoupling in order to translate their findings into mammals including humans. Further, the associated glucotoxicity, lipotoxicity, inflammation, oxidative stress, and hyperinsulinemia in T2DM combine to activate 5′ AMP-activated protein kinase (AMPK) and mammalian target of rapamycin complex 1 (mTORC1), which impairs mitophagy via mTORC1 to cause phosphorylation of Unc51-like kinase, hATG1 (ULK1) [101]. Additionally, there may also be impairment of autophagy-related proteins ATG/microtubule-associated proteins 1A/1B light chain LC3 (ATG/LC3) machinery in T2DM [102]. This allows the accumulation of aMt to leak mtROS and mtDNA to promote cellular apoptosis as well as instigating inflammation. As noted in Figure 5 in the introduction Section 1, selective mitophagy is governed by Pink1/Parkin and impairment of this pathway is associated with diabetes and neurodegeneration [103].

The problem with T2DM is global, and the International Diabetes Federation has reported that the number of adults aged 18–99 years old with T2DM is 451 million strong and also predicts that T2DM may rise to 693 million in 2 more years by 2024 [104]. This increased risk for T2DM in both obese and aging populations that are coupled with the global demographics requires a better understanding of the molecular changes. This review has been an attempt to better understand the multiple pathways with a focus on ultrastructural Mt remodeling changes, which includes aMt as a nexus for the convergence of obesity, IR, and T2DM and their multiple end-organ complications [12,14,20,105,106]. While the focus in this narrative review and ultrastructural observational findings has been on aMt and impaired mitophagy allowing the accumulation of aMt, it is also important to note that there is also impaired Mt dynamics (fission and fusion) and impaired Mt biogenesis in conditions of insulin resistance in general and particularly in T2DM in addition to impaired mitophagy and the accumulation of aMt [105].

The observational TEM findings of aMt in multiple cells and organs including the brain strongly place the Mt at the very core of associating IR and T2DM and LOAD (Section 4 and Section 4.1). These findings are in strong support of a mitochondrial cascade hypothesis for LOAD as put forward by Swerdlow et al. [107]. Additionally, there are at least nine well-accepted hypotheses and three emerging hypotheses for the development of LOAD [36]. In most cases, the Mt cascade hypothesis does not exclude any of these other nine accepted hypotheses; instead, the Mt cascade hypothesis is supportive [107]. Therefore, the author remains very excited regarding the future potential of mitochondrial transfer [108]. Importantly, the intercellular transfer of Mt is a universal biological event (Section 5 and Figure 21) and indeed Mt can cross cell boundaries [68,109]. This evolving field of Mt transfer research is very exciting, and while there is a paucity of specific literature in regard to T2DM, there are papers that discuss the importance of mesenchymal stem cell therapy and artificial Mt transfer with the following references being highly recommended [108,109,110,111,112,113].

There are certain limitations to this review, in that this study only utilizes ultrastructural TEM images to demonstrate mitochondrial remodeling at a single point in time; however, one can note the marked remodeling of the aMt as compared to the mitochondria in normal control models in the images that were presented.

It has now been 70 years since George Emil Palade published the first TEM images of the mitochondria in 1952 [114] and we are still vigorously studying the mitochondrial dynamics of fusion and fission, biogenesis, and mitophagy, which are Mt processes that are impaired in obesity, IR, MetS, and T2DM. Indeed, this review has demonstrated the normal Mt phenotype in healthy control models and depicted how the remodeled aMt phenotypes in obesity, IR, MetS, and T2DM accumulate and are associated with and play a responsible central role for cellular dysfunction, damage, and apoptosis in multiple cells and organs (Figure 3 and Figure 27). It is hoped that the crossover of knowledge between the ultrastructure images presented in this review and the discipline of functional molecular biology may lead to advances in the study of mitochondria and the remodeling phenotype of aberrant Mt due to impaired mitophagy found in preclinical models of obesity, IR, MetS, and T2DM.

## Data Availability

Data and materials will be provided upon reasonable request.
